# Bioceramics in Endodontics: Updates and Future Perspectives

**DOI:** 10.3390/bioengineering10030354

**Published:** 2023-03-13

**Authors:** Xu Dong, Xin Xu

**Affiliations:** 1 State Key Laboratory of Oral Diseases and National Clinical Research Center for Oral Diseases, West China Hospital of Stomatology, Sichuan University, Chengdu 610041, China; dongxu@stu.scu.edu.cn; 2 Department of Cariology and Endodontics, West China Hospital of Stomatology, Sichuan University, Chengdu 610041, China

**Keywords:** bioceramics, endodontic diseases, vital pulp therapy, root canal therapy, endodontic microsurgery, regenerative endodontic treatment

## Abstract

Bioceramics, with excellent bioactivity and biocompatibility, have been widely used in dentistry, particularly in endodontics. Mineral trioxide aggregate (MTA) is the most widely used bioceramic in endodontics. Recently, many new bioceramics have been developed, showing good potential for the treatment of endodontic diseases. This paper reviews the characteristics of bioceramics and their applications in various clinical endodontic situations, including root-end filling, root canal therapy, vital pulp therapy, apexification/regenerative endodontic treatment, perforation repair, and root defect repair. Relevant literature published from 1993 to 2023 was searched by keywords in PubMed and Web of Science. Current evidence supports the predictable outcome of MTA in the treatment of endodontic diseases. Although novel bioceramics such as Biodentine, EndoSequence, and calcium-enriched mixtures have shown promising clinical outcomes, more well-controlled clinical trials are still needed to provide high-level evidence for their application in endodontics. In addition, to better tackle the clinical challenges in endodontics, efforts are needed to improve the bioactivity of bioceramics, particularly to enhance their antimicrobial activity and mechanical properties and reduce their setting time and solubility.

## 1. Introduction

**Figure 1:**
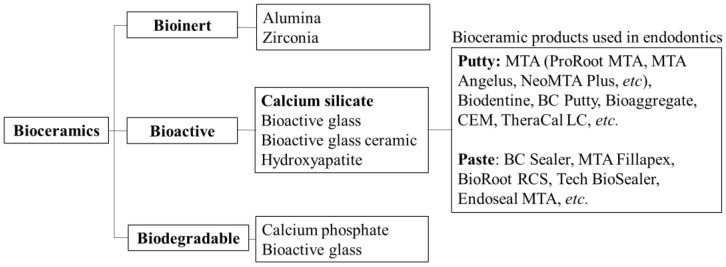
Classification of bioceramics.

In the early 1990s, bioceramics were introduced in the field of endodontics as a new group of dental materials. A mapping review of dental biomaterials found that bioceramics were a research focus between 2007 and 2019 [1]. Bioceramics are biocompatible ceramic materials or metal oxides including alumina, zirconia, bioactive glass, glass ceramics, hydroxyapatite, calcium silicate, and resorbable calcium phosphate. Bioceramics can be classified as bioinert, bioactive, and biodegradable materials based on their reactivity with surrounding tissues [2,3] (Figure 1). Bioceramics used in endodontics are generally bioactive, among which calcium silicate-based cements (CSCs) are the most common [4]. In addition to having excellent physical and chemical properties, CSCs play an important role in endodontic therapy due to their biocompatibility and bioactivity [5,6].

Over the last three decades, there has been a great deal of interest in developing bioactive dental materials that can interact and induce regeneration of the surrounding tissue. As the first bioactive ceramic material applied in endodontics, mineral trioxide aggregate (MTA) is the most studied bioceramic to date. A bibliometric study showed that MTA was a hot topic in endodontic research in the first 20 years of the 21st century [7]. MTA was developed based on Portland cement and possessed good biocompatibility and sealing abilities [8,9]. It was first introduced in dentistry as a root-end filling material in 1993 and was approved by the Food and Drug Administration (FDA) in 1997. ProRoot MTA was the first commercial MTA product launched in 1999. The first ProRoot MTA product was gray, and all subsequent products have improved on this basis. The inherent limitations of MTA include prolonged curing time, high cost, and the possibility of discoloration [10].

In the early 2000s, many modified MTA products appeared, which overcame the shortcomings of traditional MTA while retaining its original excellent performance. White MTA, which was introduced in 2002, reduced the possibility of tooth discoloration compared to gray MTA because of the lower concentrations of iron, aluminum, and magnesium oxides. MTA Angelus was launched in 2001 and was approved by the FDA in 2011. MTA Angelus has a shortened setting time and improved operability while retaining the superior performance of traditional MTA [11,12].

In the late 2000s and early 2010s, more bioceramics were developed and applied to endodontic therapy, and they have biological properties comparable to MTA, such as antibacterial activity, low cytotoxicity, and mild inflammatory response [13,14]. Products such as Biodentine, EndoSequence root repair material (ERRM), BioAggregate, and calcium-enriched mixtures (CEM) have been widely used in clinical practice [15]. Biodentine was introduced in the dental market in 2009 as a “dentine substitute”, which facilitates its penetration into open dentine tubules [16]. Biodentine is formulated using MTA-based cement technology and shows increased mechanical strength and faster solidification because it contains no calcium aluminate or calcium sulfate [17]. ERRM contains EndoSequence bioceramic putty (BC Putty) in putty form (the same as iRoot BP Plus and TotalFill RRM Putty) and EndoSequence bioceramic sealer (BC Sealer) in paste form (the same as iRoot SP and TotalFill Sealer). ERRM is a hydrophilic calcium silicate material that forms hydroxyapatite after solidification. It is a class of ready-to-use bioceramics with good operational performance and a low risk of tooth discoloration [18]. BioAggregate is an aluminum-free bioceramic and contains additives such as calcium phosphate and silica. BioAggregate has been proven to possess excellent stable bond strength and sealing properties, but relatively poor mechanical properties [19,20]. CEM, which was first applied to dentistry in 2008, is made of different calcium compounds and has similar excellent properties to MTA at a more reasonable price [21]. It has similar physical properties and clinical indications to MTA but has a different chemical composition [4]. TheraCal LC entered the market in 2011 as a light-curing resin-modified calcium silicate product for use as a liner in direct and indirect pulp-capping procedures [22].

In the last 10 years, the application of bioceramic materials in endodontics has been extensively studied. Some studies focused on the evaluation of the performance and clinical effects of existing bioceramics, while some studies focused on the update of existing bioceramics products, such as EndoSequence fast-set putty and BC Sealer HiFlow. There were also efforts to develop new bioceramics such as the tricalcium silicate-based repair material associated with 30% calcium tungstate (TCS + CaWO4), despite the lack of clinical data so far [23].

The development of various bioceramics has greatly advanced the clinical practice of endodontics. This article reviews the characteristics of bioceramics and their clinical applications in various clinical situations in endodontics, including root-end filling, root canal therapy, vital pulp therapy, apexification/regenerative endodontic treatment, perforation repair, and root defect repair. In addition, we also discuss current limitations and possible solutions to better expand the applications of bioceramics to endodontic treatment.

## 2. Search Methodology

We conducted an electronic search of relevant studies in PubMed and Web of Science databases from 1993 to 2023, with no restrictions on study type. The searched MeSH keywords included Ceramics, Dental Cements, Biocompatible Materials, and Endodontics. In addition, we manually searched major endodontics journals from the last 5 years, including the *Journal of Endodontics, International Endodontic Journal, Australian Endodontic Journal,* and *Iranian Endodontic Journal*. Reference mining was performed on the identified articles and used to locate other papers. Root-end filling, root canal therapy, vital pulp therapy, apexification, regenerative endodontic treatment, perforation repair, and root defect repair were used as keywords to locate bioceramics-related research in endodontics.

## 3. Characteristics of Bioceramics

### 3.1. Chemical Properties

In order to understand the differences between different materials, Table 1 lists the chemical composition of bioceramics used in endodontics [4,24,25,26,27]. ProRoot MTA, Biodentine, BioAggregate, and CEM are all CSCs that are composed of powder and liquid. The powder is mainly composed of dicalcium silicate and tricalcium silicate, and the main component of the liquid is water. After mixing the powder with the liquid, a mixture of mainly hydrated calcium silicate gels is produced, which eventually solidifies into a hard structure. BC Putty is a premix CSC, which is a ready-to-use material with the main components of calcium silicate and calcium phosphate. TheraCal LC is a light-cured, resin-modified calcium silicate-based paste, mainly containing type III Portland cement and resin. Both BC Sealer and EndoSeal MTA are premixed, injectable calcium silicate-based sealers, with the main difference being that EndoSeal MTA contains aluminum while BC Sealer does not. MTA Fillapex, BioRoot RCS, and Tech BioSealer are all two-component calcium silicate-based sealers whose active ingredients are MTA, tricalcium silicate, and CEM, respectively.

### 3.2. Biocompatibility and Bioactivity

The biocompatibility and bioactivity of bioceramics are mainly reflected in their interactions with surrounding tissues. Bioceramics affect the proliferation, differentiation, migration, and apoptosis of stem cells, osteoblasts/osteoclasts, dental pulp cells (DPCs)/periodontal ligament cells (PDLCs), and immune cells [6]. The response of cells to bioceramics determines the outcome of wound healing and tissue repair.

Mesenchymal stem cells (MSCs) derived from dental tissue include dental pulp stem cells (DPSCs), stem cells from human exfoliated deciduous teeth (SHED), and stem cells from apical papilla (SCAPs) [28]. MSCs have self-renewal and multidirectional differentiation potential, which is of great significance for pulp regeneration and osteogenesis [29]. Bioceramics significantly promote the attachment and survival of stem cells, and their effect on stem cells depends on cell type [28,30]. Biodentine, NeoMTA Plus, and TheraCal LC have good biocompatibility and can induce odontogenic/osteogenic differentiation of MSCs [31]. MSCs can be used in bone regeneration and tissue engineering when combined with calcium phosphate bioceramics [32]. ProRoot MTA and Biodentine show biological characteristics conducive to DPSCs activity in vitro [33]. Biodentine induces odontoblastic differentiation of DPSCs through mitogen-activated protein kinase (MAPK) and calcium-/calmodulin-dependent protein kinase II (CaMKII) pathways [34]. MTA-HP and ERRM promote the proliferation, mineralization, and attachment of DPSCs [35]. MTA and ERRM possess good biocompatibility and osteogenic properties, which promote the proliferation, adhesion, and migration of SHED [36]. MTA, Biodentine, and ERRM have shown good cytocompatibility and bioactivity when cultured with SHED [37]. ProRoot MTA, Biodentine, and ERRM can potentially induce SCAPs mineralization and odontogenic/osteogenic differentiation, supporting their application in pulp regeneration [38,39]. SCAPs co-cultured with ProRoot MTA and Biodentine showed higher adhesion ability and viability than BioRoot RCS and calcium hydroxide [40]. BC Sealer significantly enhances the cell migration of SCAPs and promotes the activity of alkaline phosphatase and the formation of mineralized nodules [41].

The repair of the bone tissue around damaged teeth depends on the number and balance of osteoblasts and osteoclasts [42]. When bioceramics are used in perforation repair and root-end filling, the interaction between the materials and cells is crucial for controlling inflammation and promoting wound repair [43]. MTA significantly inhibits RANKL-mediated osteoclastogenesis and osteoclast activity, thereby inhibiting bone resorption in periapical lesions [44]. BioAggregate stimulates osteoblastic differentiation, inhibits osteoclast formation in vitro, and shows considerable inhibitory effects on osteoclastic differentiation and inflammatory bone resorption in vivo [45,46,47]. BC Sealer and ProRoot ES show better biocompatibility than conventional root canal sealers and promote osteoblastic differentiation [48].

DPCs/PDLCs are involved in wound healing and the regeneration of teeth and periapical tissues [49]. Bioceramics interact with DPCs/PDLCs when used for pulp capping, perforation repair, and root-end filling. MTA, Biodentine, BioAggregate, and ERRM induce the expression of genes related to mineralization and odontoblastic differentiation in DPCs [50,51,52,53,54]. BioAggregates also promote the adhesion, migration, and attachment of DPCs [55]. Biodentine, MTA Angelus, and ERRM have low cytotoxicity and high cell viability against DPCs in vitro and can be used as biocompatible materials in vital pulp therapy [56,57]. Bioceramics such as ProRoot MTA, Biodentine, and ERRM show favorable effects on the odontogenic differentiation of DPCs in vitro and can effectively promote the formation of high-quality dentine bridges [58]. MTA Fillapex and BC Sealer induce lower expression of inflammatory mediators and enhanced osteoblastic differentiation of PDLCs through integrin-mediated signaling pathways [59].

When a biomaterial is placed into the tissue, immune cells, such as monocytes and macrophages, respond immediately. Macrophages release proinflammatory cytokines, such as TNF-α, IL-1, and IL-12, at the onset of the acute inflammatory response; anti-inflammatory cytokines, such as IL-4, are released during tissue regeneration and healing [60,61]. MTA changes the secretion of inflammatory cytokines, participates in leukocyte recruitment and extravasation, and regulates inflammatory control and tissue healing in pulpitis and periapical diseases [62,63]. MTA and BC Sealers have good biocompatibility with macrophages, inducing M1 and M2 polarization in RAW 264.7 and promoting the release of their proinflammatory cytokines [64,65,66]. Biphasic calcium phosphate ceramics can promote the CaSR-mediated polarization of M2 macrophages for bone induction through the continuous release of calcium ions [67].

Several studies have investigated the biocompatibility and bioactivity of bioceramics in endodontics. MTA is the most thoroughly investigated material and has been considered the “gold standard”. There are not enough studies to evaluate other bioceramics compared to MTA, and there are differences in the methods and results of various in vitro models. Therefore, more comprehensive experiments are needed to provide high-level evidence for the application of these materials in endodontic treatments.

## 4. Clinical Applications in Endodontics

**Figure 2:**
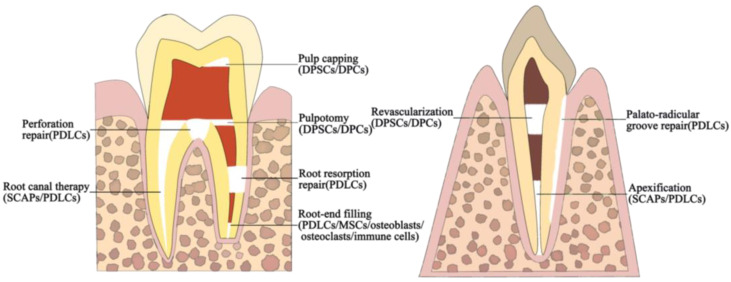
Schematic diagram of a clinical application of bioceramics in endodontics. Cells surrounding the bioceramics are shown in parentheses.

Bioceramics have been widely used in various endodontic clinical settings (Figure 2). Bioceramic putties such as MTA, Biodentine, BioAggregate, BC Putty, and CEM are commonly used for root-end filling, vital pulp therapy (VPT), apexification/regenerative endodontic therapy, perforation repair, and root defect repair. Bioceramic pastes, such as BioRoot RCS and BC Sealer, are commonly used as sealing agents in root canal fillings.

### 4.1. Root-End Filling

Root-end filling can be achieved using either orthograde or retrograde filling, both of which aim to achieve apical sealing. An ideal apical sealing material should have bioactivity, biocompatibility, long-term sealing ability, good operating performance, and the ability to promote tissue healing [8,68,69]. In dentistry, almost all available restorative materials have been used as root-end filling materials, and bioceramics such as MTA are among the most prominent [70].

#### 4.1.1. Orthograde Filling

**Figure 3:**
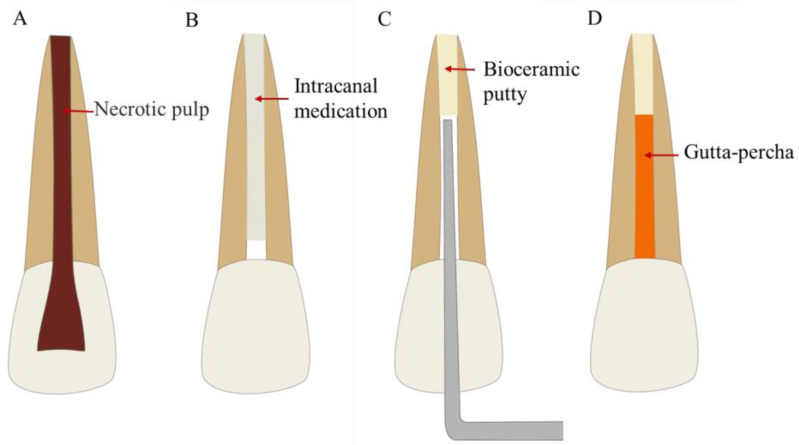
Treatment procedures for the apical barrier technique. (**A**) Permanent teeth with incompletely developed apical foramen and pulp necrosis or periapical disease. (**B**) Root canal disinfection. (**C**) Apical barrier using bioceramic putty. (**D**) Root canal filling.

Orthograde filling generally refers to the apical barrier technique, which transports MTA or other materials from the coronal side of the root canal to the apical position to seal the apex of the tooth and provide conditions for the rigorous root canal filling [71] (Figure 3). MTA has been widely used in the apical barrier technique and has achieved long-term clinical and radiographic success [72,73,74,75,76]. In a case series of 5–15 years, MTA as an apical barrier for the treatment of nonvital immature teeth achieved a healing rate of 96% [77].

The application of other bioceramics as apical barriers has also been reported. Biodentine as an apical barrier is better at preventing bacterial leakage than MTA in vitro [78,79]. Apical barrier techniques using MTA, Biodentine, and CEM increase the fracture resistance of immature teeth [80,81]. CEM as an apical barrier material has a smaller or similar amount of leakage to MTA as determined by the fluid filtration method in vitro [82,83,84]. The results of the liquid filtration show that BioAggregate and white MTA apical plugs have similar leakage resistance [85]. In a clinical trial, the 2-year success rate of 11 teeth treated with MTA and BioAggregate was 100% [86].

Of note, MTA is currently the most recommended material for apical barriers, while other materials such as Biodentine, BioAggregate, and CEM require more high-quality studies to prove their effectiveness in this clinical application.

#### 4.1.2. Retrograde Filling

Retrograde filling is a surgical method for the treatment of recurrent periapical lesions, to seal the root end and avoid the spread of infection in the root canal system [87]. Retrograde filling is performed after 3 mm of apical resection and 3 mm of root-end preparation, which is one of the most critical steps in endodontic microsurgery and intentional replantation [88,89].

##### Endodontic Microsurgery

**Figure 4:**
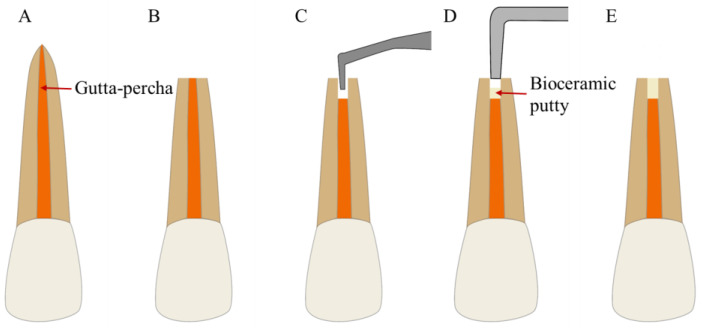
Treatment procedures for EMS. (**A**) A tooth with refractory periapical disease. (**B**) Root-end resection. (**C**) Root-end preparation. (**D**) Root-end filling with bioceramic putty. (**E**) Complete root-end filling.

Endodontic microsurgery (EMS) is an effective method for tooth preservation in patients with complicated periapical diseases. The clinical outcomes of apical surgery are inseparable from rigorous root-end filling, which is a critical step in ensuring effective apical closure to reduce microleakage and reinfection [90] (Figure 4). Bioceramics, such as MTA, are widely used in EMS because of their good biocompatibility, excellent sealing ability, inhibition of pathogenic microorganisms, and ability to promote the healing of periapical tissues [91]. The success rate of bioceramics is significantly higher than that of amalgam and resin materials and is similar to the use of intermediate repair materials (IRM) and super ethoxybenzoic acid (Super EBA) as root-end filling materials in apical surgery [91,92,93].

The success rate of 1–5 years of bioceramics as root-end filling materials in EMS is 86.4–95.6% [91]. MTA and Biodentine have splendid biocompatibility and apical sealing abilities, and both can promote periapical bone healing in vitro [94,95]. The use of fast-setting CSCs in EMS is recommended, especially in complicated clinical situations that require the rapid initial setting of materials [96]. BC Putty shows similar apical sealing performance to MTA in vitro and may better induce tissue healing adjacent to the resected root surface [97,98,99,100]. A retrospective clinical study [101] showed that the success rates of 6 months to 9 years for teeth with ProRoot MTA and BC Putty root-end filling were 92.1% and 92.4%, respectively. The one-year overall success rate of EMS using BC Putty was 92.0% in another retrospective clinical trial [102]. In prospective clinical studies, the one-year success rates of MTA and BC Putty were all greater than 93%, indicating a good prognosis [103,104].

Apical surgery was the earliest field of bioceramic application. MTA and BC Putty are well-proven root-end filling materials with predictable outcomes. However, there is insufficient evidence to conclude that any material is superior to the other [105,106]. Nonetheless, more randomized controlled trials are needed to provide high-level evidence for their effectiveness.

##### Intentional Replantation

**Figure 5:**
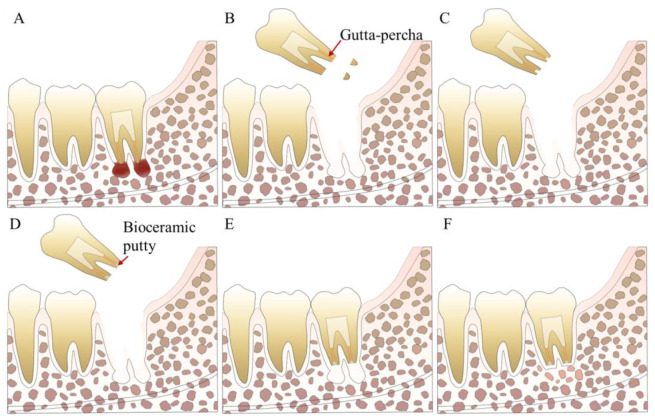
Treatment procedures for IR. (**A**) A tooth with refractory periapical disease. (**B**) Tooth extraction and root-end resection. (**C**) Root-end preparation. (**D**) Root-end filling with bioceramic putty. (**E**) Tooth replantation. (**F**) Apical inflammation disappears.

Intentional replantation (IR) is a method of extracting an intact affected tooth and replanting it in situ after treatment, which is suitable for the failure of EMS or root injury that cannot be repaired in the mouth [107] (Figure 5). Recent studies show that IR has a more consistent success rate of 88% to 95%, and it is considered a more commonly accepted therapeutic strategy [108,109]. IR is a cost-effective alternative to root canal retreatment and tooth extraction in appropriate cases [110,111,112,113,114,115]. The long-term success and survival of IR depend on numerous factors, one of which is the type of root-end filling material [116].

The application of MTA in EMS has achieved good clinical outcomes. However, some reports have argued that MTA may not achieve the same effect in IR. It was found that an extraoral time of more than 15 min and the use of ProRoot MTA as a root-end filling material in IR were significantly associated with a lower survival rate [117]. A prospective study showed that the replantation time over 15 min had a 28.6% risk of ankylosis and a 12.7% probability of persistent or emerged periapical radiolucency when retro-filled with ProRoot MTA, which significantly reduced the healing rate [118]. The long operation time of MTA and its susceptibility to blood contamination may lead to a decrease in its sealing ability and resistance to wash-out. Therefore, it is recommended to use fast-setting bioceramics for the root-end filling of IR. Many new bioceramics with good operability have been reported for IR. Good clinical results using BC Putty and CEM in IR can be seen in some case reports [119,120,121,122]. In case series, root-end filling with CEM for IR was also successful in 90% of teeth at a mean follow-up of 15.5 months [123].

There is no clear clinical treatment protocol or guideline for IR, which leads to differences in surgical procedures and the lack of specialized studies on filling materials. MTA is the most widely used material; however, its effectiveness is debatable. Case reports using BC Putty or CEM exist, but the research is of low quality. Therefore, further studies and long-term follow-up of clinical trials are required.

### 4.2. Root Canal Therapy

**Figure 6:**
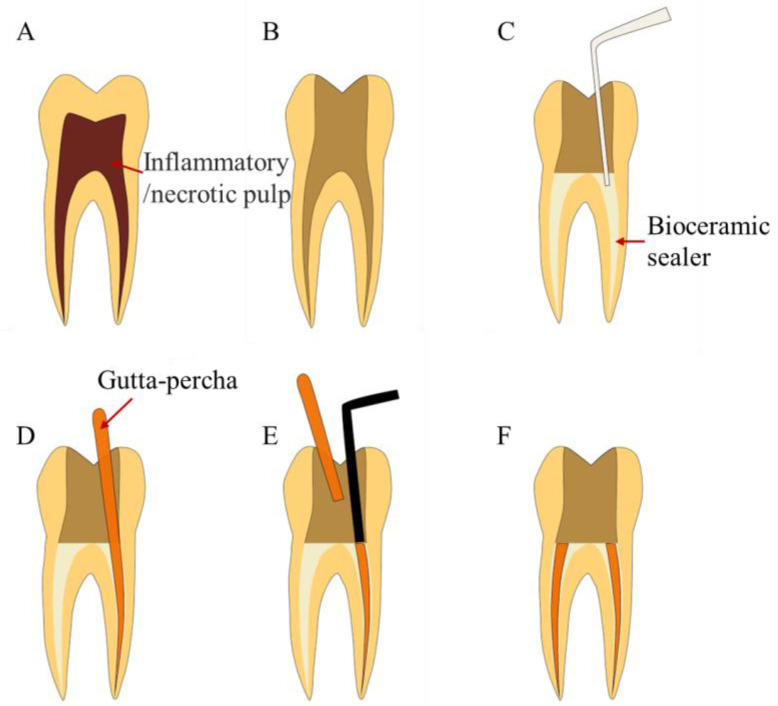
Treatment procedures for single-cone technique. (**A**) A tooth with pulpal or periapical disease. (**B**) Root canal cleaning and shaping. (**C**) Inject bioceramic sealer. (**D**) Insert gutta-percha. (**E**) Cut off the gutta-percha from the root canal orifice. (**F**) Complete root canal filling.

Root canal therapy is the most effective and most common method for treating pulpal and periapical diseases [124]. The single-cone technique is an easy-to-operate and time-saving method of root canal therapy, with sealer as the main material and gutta-percha as an auxiliary [125] (Figure 6). In addition, the GentleWave system utilizes advanced fluid dynamics to clean root canals, minimizing excessive cutting caused by mechanical preparation and reducing the risk of intracanal separation of Ni–Ti rotary instruments [126,127,128]. After root canal cleaning, hydraulic condensation with bioceramic sealer is used for root canal obturation, especially for irregular root canals. These techniques are increasingly dependent on the root canal sealer, so the fluidity and other physicochemical properties of the sealer play a crucial role in the success of treatment [129]. Bioceramic sealers, such as BC Sealer, possess good biocompatibility, superior fluidity, and chemical stability. When applied to the single-cone technique, bioceramic sealers have achieved satisfactory short-term clinical results [130,131].

The combinatory use of bioceramic sealers and the single-cone technique has achieved excellent outcomes. Root canal filling using gutta-percha/bioceramic sealer has a similar or shorter postoperative pain duration than gutta-percha/traditional sealer [132,133]. An overall success rate of 90.9% using the BC Sealer and single-cone technique was achieved from a retrospective study [134]. BC Sealer combined with the single-cone technique achieved an 88.7% success rate for initial treatment and a 63.9% success rate for retreatment in another retrospective study [135]. In prospective studies, the BioRoot RCS combined with the single-cone method has achieved a 1-year success rate of 90~97.44%, which is comparable to the 89~93.33% success rate of warm vertical condensation of gutta-percha using resin-based sealers [136,137]. A randomized clinical trial using epoxy and calcium silicate-based sealers in a single-cone technique showed no significant differences in postoperative pain or healing process [138].

Based on current evidence, the single-cone method combined with bioceramics has achieved satisfactory clinical results and has great operability. However, it is not currently accepted by most clinicians because of the lack of standardized clinical guidelines and the high reliance on root canal sealers. The use of the single-cone method remains controversial and requires long-term clinical trials with large sample sizes.

### 4.3. Vital Pulp Therapy

The treatment strategy for exposed vital pulp teeth has shifted to conservative and minimally invasive treatment, which is closely related to the development of bioactive dental materials in recent years [139,140]. VPT includes pulp capping and pulpotomy, which are methods for maintaining the vitality and function of the pulp after injury, decay, or restorative procedures [141,142]. The selection of the capping material is one of the keys to success, and MTA is a commonly used and widely studied material [24]. The American Association of Endodontics (AAE) recommends the use of CSCs in VPT, whose clinical application has been consistently successful [143].

#### 4.3.1. Pulp Capping

**Figure 7:**
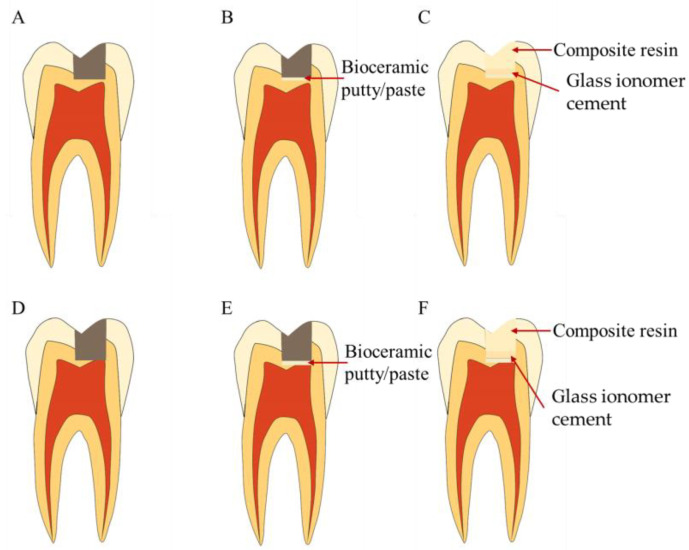
Treatment procedures for pulp capping. (**A**–**C**) Treatment procedures for indirect pulp capping. (**A**) Caries or defect close to the pulp. (**B**) Indirect pulp capping with bioceramic putty or paste. (**C**) Coronal filling. (**D**–**F**) Treatment procedures for direct pulp capping. (**D**) Caries or defect in contact with the pulp. (**E**) Direct pulp capping with bioceramic putty or paste. (**F**) Coronal filling.

Pulp capping refers to covering the dentin surface close to the pulp or covering an exposed pulp wound with a repair material to protect the pulp and eliminate the lesions [144]. Pulp capping can be divided into direct pulp capping (DPC) and indirect pulp capping (IPC), depending on whether the material is in direct contact with pulp tissue [140,145] (Figure 7). The application of MTA in DPC has been the most studied topic. MTA used in DPC can achieve predictable clinical outcomes and is more effective in maintaining the long-term viability of the pulp than calcium hydroxide [146,147,148,149,150].

Other bioceramics have been reported for pulp capping. BioAggregate has excellent cellular compatibility in vitro and is a possible alternative to MTA for pulp capping [55]. BC Putty also has comparable biocompatibility with MTA for pulp tissue and can induce the formation of restorative dentin bridge [151,152,153]. Biodentine and MTA Angelus lead to satisfactory results in vitro, showing a light inflammatory response and pronounced barrier formation for mineralization [154]. The dentin bridge formation thickness of Biodentine is higher than that of CEM and MTA in a clinical study, but it shows greater pulp inflammation [155]. Biodentine has better clinical and histological performance as a DPC agent compared with Dycal (a calcium hydroxide-based product), as demonstrated by reduced postoperative pain and sensitivity, thicker dentin bridge formation, and less pulpal inflammation [156,157]. Biodentine, with its high operability and competitive price, has no distinguishing success rate for DPC in 1–3 years compared to MTA [158,159,160,161,162,163].

Based on available evidence, bioceramics promote reliable mineralized tissue formation and sustained pulp vitality. MTA and Biodentine are currently the most studied materials and are recommended for pulp capping. Although other bioceramics (such as BC Putty and CEM) have been studied less, they have also achieved better results than traditional calcium hydroxide.

#### 4.3.2. Pulpotomy

**Figure 8:**
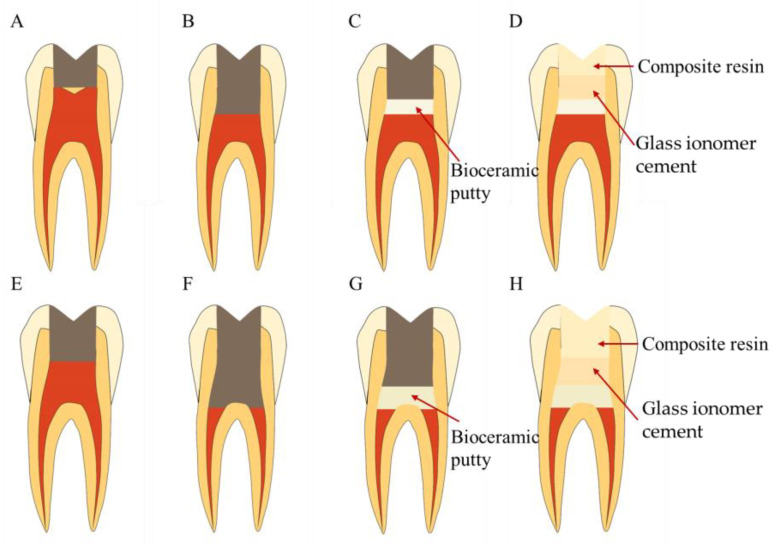
Treatment procedures for pulpotomy. (**A**–**D**) Treatment procedures for partial pulpotomy. (**A**) Caries or defect in contact with the pulp. (**B**) Removal of part of the coronal pulp. (**C**) Pulp capping with bioceramic putty. (**D**) Coronal filling. (**E**–**H**) Treatment procedures for complete pulpotomy. (**E**) Caries or defect in contact with the pulp. (**F**) Removal of all the coronal pulp. (**G**) Pulp capping with bioceramic putty. (**H**) Coronal filling.

Pulpotomy is a method to remove inflamed pulp tissue and cover the pulp section with a pulp-capping agent to retain healthy pulp tissue [164]. Pulpotomy can be divided into partial and complete pulpotomy according to the depth of pulp resection [165] (Figure 8). The application of MTA in pulpotomy can achieve outstanding results, which is supported by high-quality evidence [166,167,168,169,170,171]. Studies have shown that MTA has a better success rate than calcium hydroxide in mature permanent teeth undergoing partial pulpotomy [172,173,174].

The use of Biodentine in pulpotomy results in a success rate similar to that of MTA and reduces the likelihood of discoloration [175,176]. A prospective randomized controlled trial gave evidence that MTA and Biodentine used in pulpotomy have 100% and 89.4% success probabilities after 2 years, respectively [177]. Prospective studies showed a one-year success rate of 95–98.4% for total pulpotomy with Biodentine in mature permanent teeth with irreversible pulpitis [178,179]. Additionally, pulpotomy using hydraulic calcium silicate cements (HCSCs) has an 81–90% radiological success rate [180]. BC Putty shows a good response to partial pulpotomy in clinical cases, and it may be an effective covering material for the pulpotomy of young permanent teeth after trauma [181,182,183]. Total pulpotomy with BC Putty successfully treated 90.5% of permanent teeth with irreversible pulpitis in a prospective cohort study [184]. In clinical trials using CEM and MTA for the pulpotomy of vital immature permanent molars, all cases (49 teeth) showed pulp survival and signs of continuous root development after 1 year [185]. Randomized controlled trials have found that MTA and CEM are equally effective pulpotomy agents in mature permanent teeth of different age groups, with a 5-year success rate of over 98% [186]. Pulpotomy used with MTA/CEM is recommended as a viable and favorable alternative to root canal therapy in mature permanent teeth, demonstrating considerable and effective postoperative pain relief [187,188,189,190,191,192,193].

Although root canal therapy is still the current standard treatment for mature permanent teeth with irreversible pulpitis, the advent of bioceramics makes pulpotomy an effective alternative [164]. The determination of strict indications is necessary, and randomized clinical trials with sufficient sample sizes and long-term follow-up are still needed for further comparison of the two treatments [194,195]. Based on the current evidence, MTA is still the first choice for pulpotomy, although bioceramics such as Biodentine, BC Putty, and CEM also have great potential.

### 4.4. Apexification and Regenerative Endodontic Treatment

Since dental stem cells can promote root development, some strategies are used to treat young permanent teeth with pulp necrosis but incomplete root development [196,197,198]. Apexification and regenerative endodontic therapy are effective options for periapical tissue healing and open apical closure [199,200,201,202]. In addition to dental stem cells, biomaterials are also key factors in therapy [203].

#### 4.4.1. Apexification

**Figure 9:**
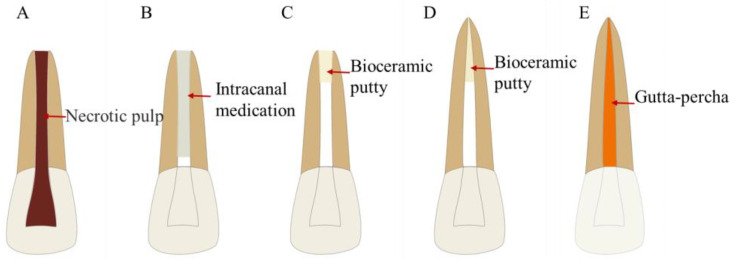
Treatment procedures for apexification. (**A**) Permanent teeth with incompletely developed apical foramen and pulp necrosis or periapical disease. (**B**) Root canal disinfection. (**C**) Apexification with bioceramics. (**D**) Root development. (**E**) Root canal filling.

Apexification refers to the placement of drugs in the root canal, which causes the root to continue to develop, and the apical foramen to narrow or close [204] (Figure 9). Compared with calcium hydroxide, MTA used in apexification induces better apical closure and less inflammatory infiltration and reduces the frequency of treatment and the possibility of tooth fracture [205,206,207].

Many new bioceramics have been reported for apexification [208]. Biodentine and ProRoot MTA prevent early root fractures during the first 30 days of apexification, and this effect is superior to that of NeoMTA Plus [209]. Several cases used Biodentine in apexification and suggested that it might increase the resistance of immature teeth [210,211,212,213,214,215]. A randomized clinical trial showed that using Biodentine in the apexification of nonvital immature molars achieved good apical healing comparable to MTA and reduced treatment time [216]. There is no difference in the amount of leakage measured by the glucose leakage model when MTA and BC Putty are used for apexification in vitro [217]. However, there are also studies in which the leakage of MTA is less than that of BC Putty measured using the radioactive isotope method in the apexification model [218]. BC Putty also promotes the continued maturation and development of immature teeth with nonvital pulp [219]. The clinical success rates of BC Putty, MTA, and calcium hydroxide are similar; however, the former two materials require a shorter time for the formation of an apical barrier and only need a single visit [220].

MTA is currently recommended as the first-choice treatment for apexification. Biodentine, BC Putty, and other materials used for apexification almost be seen in case reports. Therefore, more high-quality assessments are needed in the future.

#### 4.4.2. Regenerative Endodontic Treatment

**Figure 10:**
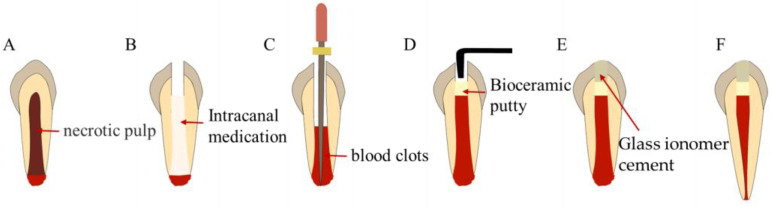
Treatment procedures for revascularization. (**A**) Immature necrotic permanent teeth. (**B**) Root canal disinfection. (**C**) Stimulate blood clot formation. (**D**) Fill with bioceramic putty. (**E**) Coronal seal. (**F**) Root development.

Regenerative endodontic treatment (RET) is an alternative to apexification in suitably selected cases and shows better results than apexification in increasing root thickness and length [199,221]. Blood clot induction, also known as revascularization, is a commonly used RET technique. Revascularization stimulates blood clots in the periapical tissues of teeth after removing the infection in the root canal by disinfection, which recruits stem cells around the root to proliferate, differentiate, and promote the formation of “new pulp tissues” in the root canal [222,223] (Figure 10). MTA is the most widely applied sealing material in RET and has an excellent overall survival rate [224,225].

The sealing material for revascularization is in direct contact with the blood clot, and this is why it is required to be bioactive, biocompatible, noncytotoxic, and antimicrobial [226]. New bioceramics are strong candidates for the coronal sealing of previously established blood clot stents. Biodentine, ProRoot MTA, and RetroMTA induce the proliferation of SCAPs, which can be used as effective sealing materials for RET [227]. Biodentine promotes the release of transforming growth factor-beta 1 (TGF-β1) from the root canal dentin and leads to higher mineralization of human apical papilla cells (APC) than ProRoot MTA [228]. MTA and Biodentine used for RET show similar void characteristics and tortuosity and there are no differences in sealing ability in vitro [229]. Biodentine has been used as a barrier material for RET, with good results in some case reports [230,231,232]. RET using bioceramic putty can result in partial or complete apical closure at an average of 54.4 months [233]. BC Putty and MTA used in RET result in apical healing and root maturation in 75% of teeth, which is thought of as a viable treatment option [234].

The level of evidence for the use of bioceramics other than MTA in RET is low, as it is generally seen in in vitro studies and case reports [235]. RET is a future direction for pulp necrosis in immature teeth, and more high-quality studies are needed to support it with the development of bioceramics.

### 4.5. Perforation Repair

**Figure 11:**
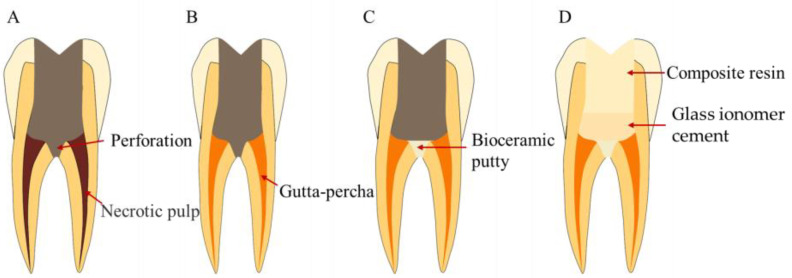
Treatment procedures for perforation repair. (**A**) Perforation in the floor of the pulp chamber. (**B**) Root canal therapy. (**C**) Perforation repair with bioceramics. (**D**) Coronal filling.

Tooth perforation is the connection between the wall of the root canal and periodontal space [236]. The repair of perforation by bioactive nonabsorbable materials is the key to treatment (Figure 11). The three most widely recommended materials for sealing root perforations are calcium hydroxide, MTA, and CSCs [237]. MTA is the standard material for the repair of furcal perforations and can produce a favorable histological response [238]. NeoMTA Plus shows better early biocompatibility than MTA Angelus, EndoSeal MTA, and ProRoot MTA, providing similar sealing ability [239,240].

Other bioceramics have also been used for perforation repairs. Biodentine and MTA result in similar periradicular inflammatory responses and bone resorption when they are used to seal perforations [241,242]. When used for sealing the furcal perforation, Biodentine is more effective in preventing dye leakage than MTA [243,244,245,246]. Biodentine and MTA can reduce the risk of potentially harmful stress in the perforation region [247]. BC Putty used in repairing furcation perforations shows similar and even less leakage to MTA in vitro [248,249]. CEM and Portland cement are used to repair furcal perforation, and their ability to prevent dye and bacterial leakage is similar to MTA [250,251,252]. Premixed bioceramics are promising materials for repairing furcal perforations in primary molars, with better sealing performance and clinical outcomes than MTA [253].

Although data on the long-term efficacy of MTA in the treatment of perforation are scarce, available evidence suggests that MTA has a great sealing ability [254,255]. Biodentine, BC Putty, and others have shown similar and even better sealing performances in perforation repair than MTA in vitro. However, there are only a few clinical studies on these materials, and more high-quality studies are required to evaluate their clinical applications.

### 4.6. Root Defect Repair

Root defects such as the palate–radicular groove and root resorption are intractable diseases with a poor prognosis, and various surgical and nonsurgical methods are used to repair them [256]. Bioceramics are often preferred because the materials may directly contact the tooth and periodontal and apical tissues [257].

#### 4.6.1. Palatal-Radicular Groove

**Figure 12:**
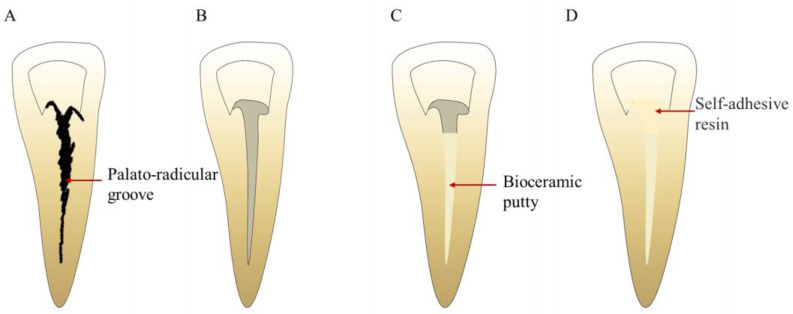
Treatment procedures for PRG repair. (**A**) PRG. (**B**) PRG preparation. (**C**) PRG filling with bioceramic putty. (**D**) Cervical filling.

The palatal-radicular groove (PRG) is defined as a developed groove in the root, usually located on the palatal side of the maxillary incisors [258] (Figure 12). PRG is a developmental abnormality, most likely due to genetic factors [259]. PRG must be filled to block the infection pathway after cleaning and preparation, and the filling materials include glass ionomer cement (GIC), composite resin, and CSCs [260].

The mechanical properties and biocompatibility of the filling material are important considerations because PRG is distributed in both the tooth crown and root. Bioceramics have an advantage over the other materials mentioned above in this respect. MTA for PRG repair has been observed in some cases, and its poor operability and risk of teeth discoloration are major concerns [261,262,263]. Moreover, Biodentine has been used to seal PRG to achieve long-term preservation of affected teeth with combined periodontal lesions in some cases [264,265,266,267]. IR for PRG of maxillary incisors has also been reported, in which BC Putty was used to fill the PRG [268,269].

PRG-related studies are limited to case reports. There are no in vitro studies and prospective clinical studies, and even fewer studies on filling materials for PRG. With the development of bioactive materials, it is hoped that more materials can be applied to the study of PRG to provide a basis for treatment.

#### 4.6.2. Root Resorption

**Figure 13:**
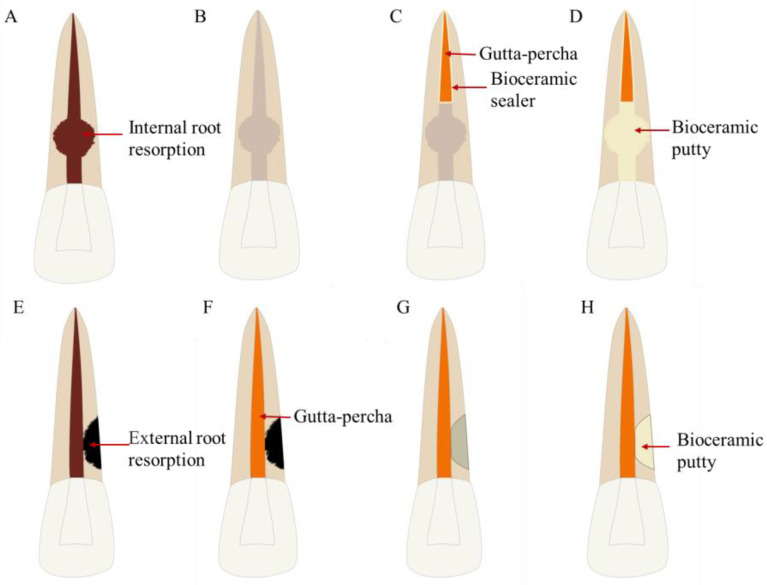
Treatment procedures for root resorption repair. (**A**–**D**) Treatment procedures for internal root resorption repair. (**A**) Internal root resorption. (**B**) Root canal cleaning. (**C**) Root canal filling in the apical segment with gutta-percha and bioceramic sealer. (**D**) Internal root resorption repaired with bioceramic putty. (**E**–**H**) Treatment procedures for external root resorption repair. (**E**) External root resorption. (**F**) Root canal therapy. (**G**) External root resorption preparation. (**H**) External root resorption repaired with bioceramic putty.

Root resorption, which can be simply divided into internal and external resorption, refers to the loss of dental tissue on the inner or outer surfaces [270,271]. The management of root resorption can include conservative or surgical treatment, depending on the location, degree, and extent of occurrence [272] (Figure 13). Root resorption and perforation appear together in many cases, and MTA used in their treatment has been reported to have satisfactory long-term results [273,274,275,276,277,278,279,280].

Bioceramics other than MTA have been reported in some cases. BC Putty, MTA, and Biodentine provide higher fracture resistance to the teeth when filling the internal resorption compared with the gutta-percha/sealer technique [281,282]. Moreover, Biodentine and CEM used in the treatment of tooth absorption have shown good results in case reports [283,284,285,286,287,288,289]. Nonsurgical repair using bioceramic putty is an effective treatment option for external cervical resorption [290]. Bioceramic sealers (MTA Fillapex and BC Sealer) show high PH values, calcium release, and good root strengthening potential, and have the potential to repair root absorption defects with satisfactory results [270,291].

Cases of root resorption are rare and complicated, and we find that all related clinical studies are case reports rather than prospective trials. MTA has been used to repair root resorption defects in many cases, and other bioceramics, such as Biodentine, CEM, and BC Putty, also have a certain degree of application. As there is no comparative study on filling materials, the selection can only be made according to the situation of specific cases.

## 5. Perspectives

To date, MTA has been the most studied bioceramic in endodontics. MTA has been demonstrated to have a predictable clinical outcome in the treatment of endodontic diseases and has been recognized as the gold standard for the development of novel bioceramics. Currently, various novel bioceramics have been developed, aiming to improve their physical and chemical properties and to reduce technique sensitivity and potential tooth discoloration. Comparable biocompatibility and bioactivity as well as clinical outcomes of various novel bioceramics have been reported. However, the antimicrobial activity, mechanical properties, setting time, and solubility of bioceramics need to be improved in the future.

Bacteria are the main cause of endodontic diseases. Antimicrobial properties are an important prerequisite for the application of bioceramics in endodontics. However, only a few bioceramics have been proven to have potent antimicrobial activity against intracanal biofilms [292]. Recent progress in bioceramic-based scaffolds with antibacterial activity includes drug-induced, ion-mediated, and physically activated, and their combined antibacterial strategies are according to the specific antibacterial mechanism [293]. Doping antibacterial ions, such as silver, copper, and zinc ions into bioceramic scaffolds can improve their anti-infection activity. Silver (Ag) is one of the best-known antibacterial agents and can be introduced in a variety of forms into different bioceramics. Incorporating silver ions into hydroxyapatite (HA) results in excellent antibacterial activities against *Pseudomonas aeruginosa* [294]. Consistently, β-tricalcium phosphate (β-TCP) augmented with silver as a bone grafting material may minimize potential infections [295]. Copper (Cu) is a commonly used therapeutic agent with remarkable angiogenic and antimicrobial activities, and the release of Cu^2+^ can be controlled by clever design and effective methods. Cu^2+^ is introduced to silicon-containing bioceramics to simultaneously enhance their mechanical and antibacterial properties [296]. Zinc (Zn) shows osteogenic, angiogenic, and antibacterial properties. Bioactive glass scaffolds containing Zn^2+^ exhibit cytocompatibility and antibacterial abilities [297]. In addition, the introduction of antibiotics or drugs into bionic bone scaffolds and the use of bioceramics and scaffolds to control their release can increase the antibacterial activity. Lactic-co-glycolic acid (PLGA)-coated chitosan microspheres loaded with HA and doxycycline hyclate complexes have been developed for periodontal delivery [298]. Endodontic sealers that incorporate novel, highly loaded antimicrobial drug-silica coassembled particles (DSPs) show great antimicrobial activity [299]. The physical antibacterial function of bioceramics is another important strategy. Nanomaterials and nanostructures have unique physical and chemical properties that may physically activate antibacterial activity, particularly against drug-resistant bacteria [300]. Therefore, traditional bioceramics in endodontics are expected to be improved by adding ions, loading antibiotics, and activating nanomaterials to address the challenges of infection control in endodontics.

Good mechanical properties are critical in certain clinical procedures of endodontics; however, bioceramics nowadays have not achieved adequate requirements [3]. Further research is required to improve the mechanical properties of bioceramics without altering their biological activity. To date, various approaches have been adopted to strengthen the mechanical properties of calcium phosphate scaffolds, including scaffold structural optimization, ink modification, sintering optimization, and the fabrication of ceramic-polymer composite scaffolds [301]. Calcium phosphate silicate (CPS) is a promising bioceramic for bone grafting, and iron (Fe) is a promising element that can enhance the mechanical strength of CPS ceramics [302]. Iron-doped akermanite ceramic is a suitable formulation for future bone substitute materials because it provides sufficient mechanical strength as well as good bioactivity [303]. Three-dimensional (3D) printing has provided new vitality for the manufacture of bioceramic scaffolds as it can achieve adjustable porosity and complex shape design. 3D printing-based calcium silicate bioceramic scaffolds with appropriate pore dimensions are promising for promoting mechanical properties [304]. Polymer-bioceramic composites are bone-tissue-engineering scaffolds that combine bioceramics with biocompatible polymers. The mechanical properties of bioceramics can be improved using this method. Introducing silica-based bioglass allows HA-based bioceramics to maintain a high compressive strength [305]. Polyether-ether-ketone (PEEK) is reinforced with bioactive silicate-based bioceramics as nanofillers, which exhibit significantly improved elastic modulus, flexural strength, and microhardness [306]. Iron doping, 3D printing, and polymer composites are the mainstay methods for enhancing the mechanical properties of bioceramics, which are expected to be used in endodontics with good outcomes.

When bioceramics are used for root-end filling, they are immediately in contact with the blood. Therefore, the ability to resist wash-out is an important factor that determines the sealing performance in this clinical situation. Setting time is a key factor in resisting the wash-out of bioceramics [15,307]. Currently, some fast-set bioceramics have been developed, such as EndoSequence fast-set putty and iRoot FS [308]. It is important to further optimize the solidification time of bioceramics for endodontic use. Nanomaterials, such as multiwalled carbon nanotubes (MWCNTS), titanium carbide (TC), or boron nitride (BN), can be incorporated into BioRoot RCS to shorten its setting time [309]. MTA Repair HP with a nanostructure can achieve both fast-setting and efficient bioactive activity [310]. Adding ions is a common method of material modification that can be used in bioceramics to improve their solidification properties. Calcium silicates doped with zinc and magnesium have been synthesized by the sol–gel method, showing a significant decrease in setting time compared to white MTA [311]. HPO_4_^2-^ ions are substituted in calcium sulfate dihydrate crystals during setting and have profound effects on the rheological properties and setting of the CSC paste [312]. Bi_2_O_3_ as a popular radiopacifier can prolong the initial and final setting times and retard the degree of hydration [313]. Therefore, the selection of a radiopacifier such as barium titanate (BT), which does not affect the curing time, is also a strategy to reduce the setting time of bioceramics. Most importantly, the self-setting nature of bone cement is not compromised by BT incorporation [314]. The addition of nanomaterials and ions and the replacement of the components that affect solidification are promising strategies to reduce the setting time of bioceramics, and thus may promote its application in endodontics.

The high solubility of the bioceramic materials is also a concern, as it may result in gaps between the dentinal wall and filling material, which compromise the quality of the seal. Calcium silicate-based sealers are associated with significantly higher solubility than epoxy-resin sealers (AH Plus) [315,316,317,318]. Ionic doping is a promising strategy to compensate for the shortcomings (high solubility) of bioceramic materials. Y_2_O_3_ and CeO_2_-doped SiO_2_–SrO–Na_2_O glass ceramics can release less Si^4+^ and Na^+^ [319]. The addition of nano-phase materials to bioceramics may have the potential to improve the physicochemical, microstructure, and compressive strength properties. Lower solubility composites are obtained by adding nanomaterials such as MWCNTs, TC, or BN to BioRoot RCS [309,320]. The addition of ions and nanomaterials is expected to reduce the solubility of bioceramics, thereby improving the sealing property of bioceramics in endodontics.

Commercially available bioceramics are composed of a variety of compounds, and even for the same material, the chemical composition may vary slightly depending on the manufacturer. Currently, most of the comparative studies on different bioceramic materials used commercial products. Laboratory studies with active compounds are still needed to yield consistent results. More well-controlled laboratory and clinical studies are still needed to better demonstrate the structure–function relationship of various bioceramics, which is of great significance in promoting the development of materials, and this is also a field that needs future efforts.

## 6. Conclusions

Bioceramics such as MTA have been demonstrated to possess excellent bioactivity and biocompatibility, and have been widely used in the clinical practice of endodontics. However, none of the bioceramic materials is completely ideal, and they always have their individual limitations in practical applications. With the development of materials, more and more bioceramics other than MTA have been developed, such as Biodentine, ERRM, BioAggregate, CEM, and BioRoot RCS. These new materials are used in root-end filling, root canal therapy, VPT, apexification/RET, perforation repair, and root defect repair. They have been proven to have comparable or even better clinical outcomes than MTA through numerous clinical trials, in vitro experiments, and case reports. However, high-quality clinical studies with long-term follow-ups and well-controlled laboratory studies are still scarce. To use these bioceramics with more confidence in the clinical practice of endodontics, more high-quality research evidence is needed in the future. Bioceramics play an important role in the treatment of endodontic diseases and have broad development prospects. We expect that more new or improved bioceramics will be developed in the future.

## Figures and Tables

**Table 1:** Composition of bioceramic products used in endodontics.

Bioceramics	Chemical Composition	
ProRoot MTA (gray)	Powder	Dicalcium silicate, tricalcium silicate, tricalcium aluminate, calcium sulfate, bismuth oxide, and calcium aluminoferrite
	Liquid	Sterile water
ProRoot MTA (white)	Powder	Dicalcium silicate, tricalcium silicate, tricalcium aluminate, calcium sulfate, and bismuth oxide
	Liquid	Sterile water
Biodentine	Powder	Tricalcium silicate, dicalcium silicate, calcium carbonate, calcium oxide, zirconium oxide, and iron oxides
	Liquid	Water, calcium chloride, and hydrosoluble polymer
BC Putty	Putty	Calcium silicates, monobasic calcium phosphate, zirconium oxide, tantalum oxide, proprietary fillers, and thickening agents
BioAggregate	Powder	Tricalcium silicate, dicalcium silicate, calcium phosphate, calcium hydroxide, hydroxyapatite, silicon dioxide, and tantalum oxide
	Liquid	Deionized water
CEM	Powder	Calcium oxide, sulfur trioxide, phosphorous pentoxide, silicon dioxide, trace amounts of aluminum trioxide, sodium oxide, magnesium oxide, and chloride
	Liquid	Water-based solution
TheraCal LC	Paste	Type III Portland cement, Sr glass, fumed silica, barium sulfate, barium zirconate, a resin containing bisphenol A glycidyl methacrylate (Bis-GMA), and poly dimethacrylate (PEGDMA)
BC Sealer	Paste	Calcium silicates, calcium phosphate, zirconium oxide, tantalum oxide, and thickening agents
MTA Fillapex	Paste A	Salicylate resin, bismuth trioxide, and fumed silica
	Paste B	Fumed silica, titanium dioxide, MTA (40%), and base resin
BioRoot RCS	Powder	Tricalcium silicate, zirconium oxide, and povidone
	Liquid	Water, calcium chloride, and hydrosoluble polymer
Tech BioSealer	Powder	CEM, calcium sulfate, calcium chloride, bismuth oxide, and montmorillonite
	Liquid	Dulbecco’s phosphate-buffered saline (DPBS)
EndoSeal MTA	Paste	Calcium silicates, calcium aluminates, calcium aluminoferrite, calcium sulfates, radiopacifier, and thickening agent

## References

[1] Iftikhar S., Jahanzeb N., Saleem M., Ur Rehman S., Matinlinna J.P., Khan A.S. (2021). The trends of dental biomaterials research and future directions: A mapping review. Saudi Dent J..

[2] Hench L.L. (1993). Bioceramics—From Concept To Clinic. Am. Ceram. Soc. Bullet..

[3] Raghavendra S.S., Jadhav G.R., Gathani K.M., Kotadia P. (2017). Bioceramics in endodontics—A review. J. Istanb. Univ. Fac. Dent..

[4] Dawood A.E., Parashos P., Wong R.H.K., Reynolds E.C., Manton D.J. (2017). Calcium silicate-based cements: Composition, properties, and clinical applications. J. Investig. Clin. Dent..

[5] Jitaru S., Hodisan I., Timis L., Lucian A., Bud M. (2016). The use of bioceramics in endodontics—Literature review. Clujul. Med..

[6] Song W., Li S., Tang Q., Chen L., Yuan Z. (2021). In vitro biocompatibility and bioactivity of calcium silicate-based bioceramics in endodontics (Review). Int. J. Mol. Med..

[7] Khan A.S., Ur Rehman S., Ahmad S., AlMaimouni Y.K., Alzamil M.A.S., Dummer P.M.H. (2021). Five decades of the International Endodontic Journal: Bibliometric overview 1967-2020. Int. Endod. J..

[8] Parirokh M., Torabinejad M. (2010). Mineral trioxide aggregate: A comprehensive literature review—Part I: Chemical, physical, and antibacterial properties. J. Endod..

[9] Torabinejad M., Parirokh M. (2010). Mineral trioxide aggregate: A comprehensive literature review—Part II: Leakage and biocompatibility investigations. J. Endod..

[10] Parirokh M., Torabinejad M. (2010). Mineral trioxide aggregate: A comprehensive literature review—Part III: Clinical applications, drawbacks, and mechanism of action. J. Endod..

[11] Koulaouzidou E.A., Economides N., Beltes P., Geromichalos G., Papazisis K. (2008). In vitro evaluation of the cytotoxicity of ProRoot MTA and MTA Angelus. J. Oral Sci..

[12] Lolayekar N., Bhat S.S., Hegde S. (2009). Sealing ability of ProRoot MTA and MTA-Angelus simulating a one-step apical barrier technique—An in vitro study. J. Clin. Pediatr. Dent..

[13] de Oliveira N.G., de Souza Araújo P.R., da Silveira M.T., Sobral A.P.V., Carvalho M.V. (2018). Comparison of the biocompatibility of calcium silicate-based materials to mineral trioxide aggregate: Systematic review. Eur. J. Dent..

[14] Jerez-Olate C., Araya N., Alcántara R., Luengo L., Bello-Toledo H., González-Rocha G., Sánchez-Sanhueza G. (2021). In vitro antibacterial activity of endodontic bioceramic materials against dual and multispecies aerobic-anaerobic biofilm models. Aust. Endod. J..

[15] Zafar K., Jamal S., Ghafoor R. (2020). Bio-active cements-Mineral Trioxide Aggregate based calcium silicate materials: A narrative review. J. Pak. Med. Assoc..

[16] Kaur M., Singh H., Dhillon J.S., Batra M., Saini M. (2017). MTA versus Biodentine: Review of Literature with a Comparative Analysis. J. Clin. Diagn. Res..

[17] Rajasekharan S., Martens L.C., Cauwels R., Anthonappa R.P. (2018). Biodentine™ material characteristics and clinical applications: A 3 year literature review and update. Eur. Arch. Paediatr. Dent..

[18] Eren S.K., Örs S.A., Aksel H., Canay Ş., Karasan D. (2022). Effect of irrigants on the color stability, solubility, and surface characteristics of calcium-silicate based cements. Restor. Dent. Endod..

[19] Saghiri M.A., Nazari A., Garcia-Godoy F., Asatourian A., Malekzadeh M., Elyasi M. (2013). Mechanical response of dental cements as determined by nanoindentation and scanning electron microscopy. Microsc. Microanal..

[20] El Sayed M., Saeed M. (2012). In vitro comparative study of sealing ability of Diadent BioAggregate and other root-end filling materials. J. Conserv. Dent..

[21] Asgary S., Shahabi S., Jafarzadeh T., Amini S., Kheirieh S. (2008). The properties of a new endodontic material. J. Endod..

[22] Arandi N.Z., Rabi T. (2018). TheraCal LC: From Biochemical and Bioactive Properties to Clinical Applications. Int. J. Dent..

[23] Queiroz M.B., Inada R.N.H., Jampani J.L.A., Guerreiro-Tanomaru J.M., Sasso-Cerri E., Tanomaru-Filho M., Cerri P.S. (2023). Biocompatibility and bioactive potential of an experimental tricalcium silicate-based cement in comparison with Bio-C repair and MTA Repair HP materials. Int. Endod. J..

[24] Parirokh M., Torabinejad M., Dummer P.M.H. (2018). Mineral trioxide aggregate and other bioactive endodontic cements: An updated overview—Part I: Vital pulp therapy. Int. Endod. J..

[25] Eskandari F., Razavian A., Hamidi R., Yousefi K., Borzou S. (2022). An Updated Review on Properties and Indications of Calcium Silicate-Based Cements in Endodontic Therapy. Int. J. Dent..

[26] Jafari F., Jafari S. (2017). Composition and physicochemical properties of calcium silicate based sealers: A review article. J. Clin. Exp. Dent..

[27] Al-Haddad A., Che Ab Aziz Z.A. (2016). Bioceramic-Based Root Canal Sealers: A Review. Int. J. Biomater..

[28] Morsczeck C., Reichert T.E. (2018). Dental stem cells in tooth regeneration and repair in the future. Expert Opin. Biol. Ther..

[29] Bhandi S., Alkahtani A., Reda R., Mashyakhy M., Boreak N., Maganur P.C., Vishwanathaiah S., Mehta D., Vyas N., Patil V. (2021). Parathyroid Hormone Secretion and Receptor Expression Determine the Age-Related Degree of Osteogenic Differentiation in Dental Pulp Stem Cells. J. Pers. Med..

[30] Orti V., Collart-Dutilleul P.Y., Piglionico S., Pall O., Cuisinier F., Panayotov I. (2018). Pulp Regeneration Concepts for Nonvital Teeth: From Tissue Engineering to Clinical Approaches. Tissue Eng. Part B Rev..

[31] Sismanoglu S., Ercal P. (2022). Effects of calcium silicate-based cements on odonto/osteogenic differentiation potential in mesenchymal stem cells. Aust. Endod. J..

[32] Millan C., Vivanco J.F., Benjumeda-Wijnhoven I.M., Bjelica S., Santibanez J.F. (2018). Mesenchymal Stem Cells and Calcium Phosphate Bioceramics: Implications in Periodontal Bone Regeneration. Adv. Exp. Med. Biol..

[33] Kim Y., Lee D., Kim H.M., Kye M., Kim S.Y. (2021). Biological Characteristics and Odontogenic Differentiation Effects of Calcium Silicate-Based Pulp Capping Materials. Materials.

[34] Luo Z., Kohli M.R., Yu Q., Kim S., Qu T., He W.X. (2014). Biodentine induces human dental pulp stem cell differentiation through mitogen-activated protein kinase and calcium-/calmodulin-dependent protein kinase II pathways. J. Endod..

[35] Abou ElReash A., Hamama H., Grawish M., Saeed M., Zaen El-Din A.M., Shahin M.A., Zhenhuan W., Xiaoli X. (2021). A laboratory study to test the responses of human dental pulp stem cells to extracts from three dental pulp capping biomaterials. Int. Endod. J..

[36] Wang J., Fangteng J.Z., Liu H. (2019). Effect of iRoot BP Plus on biological behavior of deciduous tooth pulp stem cells and human pulp stem cells. Shanghai J. Stomatol..

[37] Sanz J.L., Forner L., Llena C., Guerrero-Gironés J., Melo M., Rengo S., Spagnuolo G., Rodríguez-Lozano F.J. (2020). Cytocompatibility and Bioactive Properties of Hydraulic Calcium Silicate-Based Cements (HCSCs) on Stem Cells from Human Exfoliated Deciduous Teeth (SHEDs): A Systematic Review of In Vitro Studies. J. Clin. Med..

[38] Sanz J.L., Forner L., Almudéver A., Guerrero-Gironés J., Llena C. (2020). Viability and Stimulation of Human Stem Cells from the Apical Papilla (hSCAPs) Induced by Silicate-Based Materials for Their Potential Use in Regenerative Endodontics: A Systematic Review. Materials.

[39] Miller A.A., Takimoto K., Wealleans J., Diogenes A. (2018). Effect of 3 Bioceramic Materials on Stem Cells of the Apical Papilla Proliferation and Differentiation Using a Dentin Disk Model. J. Endod..

[40] Garrido M., Morales D., Saldías M.P., Fernández C., Villalobos V., Cerda O., Cáceres M. (2021). Cellular response of human apical papilla cells to calcium hydroxide and tricalcium silicate-based cements. BMC Oral Health.

[41] Wu L., Xue K., Hu G., Du H., Gan K., Zhu J., Du T. (2021). Effects of iRoot SP on osteogenic differentiation of human stem cells from apical papilla. BMC Oral Health.

[42] Jiang N., Guo W., Chen M., Zheng Y., Zhou J., Kim S.G., Embree M.C., Songhee Song K., Marao H.F., Mao J.J. (2016). Periodontal Ligament and Alveolar Bone in Health and Adaptation: Tooth Movement. Front. Oral Biol..

[43] Zhou Y., Wu C., Xiao Y. (2014). Silicate-based bioceramics for periodontal regeneration. J. Mater. Chem. B.

[44] Rezende T.M.B., Ribeiro Sobrinho A.P., Vieira L.Q., Sousa M., Kawai T. (2021). Mineral trioxide aggregate (MTA) inhibits osteoclastogenesis and osteoclast activation through calcium and aluminum activities. Clin. Oral Investig..

[45] Tian J., Qi W., Zhang Y., Glogauer M., Wang Y., Lai Z., Jiang H. (2015). Bioaggregate Inhibits Osteoclast Differentiation, Fusion, and Bone Resorption In Vitro. J. Endod..

[46] Zhang J., Zhu L., Peng B. (2015). Effect of BioAggregate on osteoclast differentiation and inflammatory bone resorption in vivo. Int. Endod. J..

[47] Zhang J., Zhu L., Yan P., Peng B. (2015). Effect of BioAggregate on Receptor Activator of Nuclear Factor-Kappa B Ligand-induced Osteoclastogenesis from Murine Macrophage Cell Line In Vitro. J. Endod..

[48] Giacomino C.M., Wealleans J.A., Kuhn N., Diogenes A. (2019). Comparative Biocompatibility and Osteogenic Potential of Two Bioceramic Sealers. J. Endod..

[49] Lin L.M., Rosenberg P.A. (2011). Repair and regeneration in endodontics. Int. Endod. J..

[50] Zhang S., Yang X., Fan M. (2013). BioAggregate and iRoot BP Plus optimize the proliferation and mineralization ability of human dental pulp cells. Int. Endod. J..

[51] Öncel Torun Z., Torun D., Demirkaya K., Yavuz S.T., Elçi M.P., Sarper M., Avcu F. (2015). Effects of iRoot BP and white mineral trioxide aggregate on cell viability and the expression of genes associated with mineralization. Int. Endod. J..

[52] Jung J.Y., Woo S.M., Lee B.N., Koh J.T., Nör J.E., Hwang Y.C. (2015). Effect of Biodentine and Bioaggregate on odontoblastic differentiation via mitogen-activated protein kinase pathway in human dental pulp cells. Int. Endod. J..

[53] Rathinam E., Rajasekharan S., Chitturi R.T., Martens L., De Coster P. (2015). Gene Expression Profiling and Molecular Signaling of Dental Pulp Cells in Response to Tricalcium Silicate Cements: A Systematic Review. J. Endod..

[54] Peng W., Huan Z., Pei G., Li J., Cao Y., Jiang L., Zhu Y. (2022). Silicate bioceramics elicit proliferation and odonto-genic differentiation of human dental pulp cells. Dent. Mater. J..

[55] Zhu L., Yang J., Zhang J., Peng B. (2014). A comparative study of BioAggregate and ProRoot MTA on adhesion, migration, and attachment of human dental pulp cells. J. Endod..

[56] Adıgüzel M., Ahmetoğlu F., Eldeniz A., Tekin M.G., Göğebakan B. (2019). Comparison of cytotoxic effects of calcium silicate-based materials on human pulp fibroblasts Mehmet. J. Dent. Res. Dent. Clin. Dent. Prospect..

[57] Zakerzadeh A., Esnaashari E., Dadfar S. (2017). In Vitro Comparison of Cytotoxicity and Genotoxicity of Three Vital Pulp Capping Materials. Iran. Endod. J..

[58] Emara R., Elhennawy K., Schwendicke F. (2018). Effects of calcium silicate cements on dental pulp cells: A systematic review. J. Dent..

[59] Chang S.W., Lee S.Y., Kang S.K., Kum K.Y., Kim E.C. (2014). In vitro biocompatibility, inflammatory response, and osteogenic potential of 4 root canal sealers: Sealapex, Sankin apatite root sealer, MTA Fillapex, and iRoot SP root canal sealer. J. Endod..

[60] Kou P.M., Babensee J.E. (2011). Macrophage and dendritic cell phenotypic diversity in the context of biomaterials. J. Biomed. Mater. Res. A.

[61] Gratchev A., Guillot P., Hakiy N., Politz O., Orfanos C.E., Schledzewski K., Goerdt S. (2001). Alternatively activated macrophages differentially express fibronectin and its splice variants and the extracellular matrix protein betaIG-H3. Scand. J. Immunol..

[62] Brackett M.G., Lewis J.B., Messer R.L., Lei L., Lockwood P.E., Wataha J.C. (2011). Dysregulation of monocytic cytokine secretion by endodontic sealers. J. Biomed. Mater. Res. B Appl. Biomater..

[63] Kabashima H., Nagata K., Maeda K., Iijima T. (2002). Involvement of substance P, mast cells, TNF-alpha and ICAM-1 in the infiltration of inflammatory cells in human periapical granulomas. J. Oral. Pathol. Med..

[64] Yuan Z., Zhu X., Li Y., Yan P., Jiang H. (2018). Influence of iRoot SP and mineral trioxide aggregate on the activation and polarization of macrophages induced by lipopolysaccharide. BMC Oral Health.

[65] Tu M.G., Sun K.T., Wang T.H., He Y.Z., Hsia S.M., Tsai B.H., Shih Y.H., Shieh T.M. (2019). Effects of mineral trioxide aggregate and bioceramics on macrophage differentiation and polarization in vitro. J. Formos. Med. Assoc..

[66] Zhu X., Yuan Z., Yan P., Li Y., Jiang H., Huang S. (2017). Effect of iRoot SP and mineral trioxide aggregate (MTA) on the viability and polarization of macrophages. Arch. Oral. Biol..

[67] Zhang J., Wu Q., Yin C., Jia X., Zhao Z., Zhang X., Yuan G., Hu H., Zhao Q. (2021). Sustained calcium ion release from bioceramics promotes CaSR-mediated M2 macrophage polarization for osteoinduction. J. Leukoc. Biol..

[68] Bodrumlu E. (2008). Biocompatibility of retrograde root filling materials: A review. Aust. Endod. J..

[69] Murata K., Washio A., Morotomi T., Rojasawasthien T., Kokabu S., Kitamura C. (2021). Physicochemical Properties, Cytocompatibility, and Biocompatibility of a Bioactive Glass Based Retrograde Filling Material. Nanomaterials.

[70] Chao Y.C., Chen P.H., Su W.S., Yeh H.W., Su C.C., Wu Y.C., Chiang H.S., Jhou H.J., Shieh Y.S. (2022). Effectiveness of different root-end filling materials in modern surgical endodontic treatment: A systematic review and network meta-analysis. J. Dent. Sci..

[71] Tabiyar K., Logani A. (2021). The Apical Extent of Mineral Trioxide Aggregate Apical Barrier Does not Influence the Treatment Outcome in a Nonvital Immature Permanent Anterior Tooth: A Split-Mouth Clinical Study. Eur. Endod. J..

[72] Pace R., Giuliani V., Nieri M., Di Nasso L., Pagavino G. (2014). Mineral trioxide aggregate as apical plug in teeth with necrotic pulp and immature apices: A 10-year case series. J. Endod..

[73] Annamalai S., Mungara J. (2010). Efficacy of mineral trioxide aggregate as an apical plug in non-vital young permanent teeth: Preliminary results. J. Clin. Pediatr. Dent..

[74] Holden D.T., Schwartz S.A., Kirkpatrick T.C., Schindler W.G. (2008). Clinical outcomes of artificial root-end barriers with mineral trioxide aggregate in teeth with immature apices. J. Endod..

[75] Sarris S., Tahmassebi J.F., Duggal M.S., Cross I.A. (2008). A clinical evaluation of mineral trioxide aggregate for root-end closure of non-vital immature permanent incisors in children-a pilot study. Dent. Traumatol..

[76] Van Pham K., Tran T.A. (2021). Effectiveness of MTA apical plug in dens evaginatus with open apices. BMC Oral Health.

[77] Ree M.H., Schwartz R.S. (2017). Long-term Success of Nonvital, Immature Permanent Incisors Treated with a Mineral Trioxide Aggregate Plug and Adhesive Restorations: A Case Series from a Private Endodontic Practice. J. Endod..

[78] Abbas A., Kethineni B., Puppala R., Birapu U.C., Raghavendra K.J., Reddy P. (2020). Efficacy of Mineral Trioxide Aggregate and Biodentine as Apical Barriers in Immature Permanent Teeth: A Microbiological Study. Int. J. Clin. Pediatr. Dent..

[79] Refaei P., Jahromi M.Z., Moughari A.A.K. (2020). Comparison of the microleakage of mineral trioxide aggregate, calcium-enriched mixture cement, and Biodentine orthograde apical plug. Dent. Res. J..

[80] Ok E., Altunsoy M., Tanriver M., Capar I.D., Kalkan A., Gok T. (2016). Fracture resistance of simulated immature teeth after apexification with calcium silicate-based materials. Eur. J. Dent..

[81] Nosrat A., Asgary S., Eghbal M.J., Ghoddusi J., Bayat-Movahed S. (2011). Calcium-enriched mixture cement as artificial apical barrier: A case series. J. Conserv. Dent..

[82] Tabrizizade M., Asadi Y., Sooratgar A., Moradi S., Sooratgar H., Ayatollahi F. (2014). Sealing ability of mineral trioxide aggregate and calcium-enriched mixture cement as apical barriers with different obturation techniques. Iran. Endod. J..

[83] Ayatollahi F., Tabrizizadeh M., Hazeri Baqdad Abad M., Ayatollahi R., Zarebidoki F. (2016). Comparison of Microleakage of MTA and CEM Cement Apical Plugs in Three Different Media. Iran. Endod. J..

[84] Ayatollahi F., Hazeri Baqdad Abad M., Razavi S.H., Tabrizizadeh M., Ayatollahi R., Zarebidoki F. (2017). Evaluating the Accuracy of Two Microleakage Assessment Methods for Mineral Trioxide Aggregate and Calcium-enriched Mixture Cement. Iran. Endod. J..

[85] Memiş Özgül B., Bezgin T., Şahin C., Sarı Ş. (2015). Resistance to leakage of various thicknesses of apical plugs of Bioaggregate using liquid filtration model. Dent. Traumatol..

[86] Tuloglu N., Bayrak S. (2016). Comparative evaluation of mineral trioxide aggregate and bioaggregate as apical barrier material in traumatized nonvital, immature teeth: A clinical pilot study. Niger. J. Clin. Pract..

[87] Paños-Crespo A., Sánchez-Torres A., Gay-Escoda C. (2021). Retrograde filling material in periapical surgery: A systematic review. Med. Oral Patol. Oral Cir. Bucal..

[88] Floratos S., Kim S. (2017). Modern Endodontic Microsurgery Concepts: A Clinical Update. Dent. Clin. North. Am..

[89] Jadun S., Monaghan L., Darcey J. (2019). Endodontic microsurgery. Part two: Armamentarium and technique. Br. Dent. J..

[90] Connert T., Weiger R., Krastl G. (2022). Present status and future directions—Guided endodontics. Int. Endod. J..

[91] Abusrewil S.M., McLean W., Scott J.A. (2018). The use of Bioceramics as root-end filling materials in periradicular surgery: A literature review. Saudi Dent. J..

[92] Kohli M.R., Berenji H., Setzer F.C., Lee S.M., Karabucak B. (2018). Outcome of Endodontic Surgery: A Meta-analysis of the Literature-Part 3: Comparison of Endodontic Microsurgical Techniques with 2 Different Root-end Filling Materials. J. Endod..

[93] Schutte H., van Hooft E. (2022). The unresolved contest between MTA and IRM as apical barrier material in apicoectomies. A retrospective cohort study. Ned. Tijdschr. Tandheelkd..

[94] Tang J.J., Shen Z.S., Qin W., Lin Z. (2019). A comparison of the sealing abilities between Biodentine and MTA as root-end filling materials and their effects on bone healing in dogs after periradicular surgery. J. Appl. Oral. Sci..

[95] Bani M., Sungurtekin-Ekçi E., Odabaş M.E. (2015). Efficacy of Biodentine as an Apical Plug in Nonvital Permanent Teeth with Open Apices: An In Vitro Study. Biomed. Res. Int..

[96] Kim D., Lee H., Chung M., Kim S., Song M., Kim E. (2020). Effects of fast- and slow-setting calcium silicate-based root-end filling materials on the outcome of endodontic microsurgery: A retrospective study up to 6 years. Clin. Oral. Investig..

[97] Chen I., Karabucak B., Wang C., Wang H.G., Koyama E., Kohli M.R., Nah H.D., Kim S. (2015). Healing after root-end microsurgery by using mineral trioxide aggregate and a new calcium silicate-based bioceramic material as root-end filling materials in dogs. J. Endod..

[98] Rencher B., Chang A.M., Fong H., Johnson J.D., Paranjpe A. (2021). Comparison of the sealing ability of various bioceramic materials for endodontic surgery. Restor. Dent. Endod..

[99] Leal F., De-Deus G., Brandão C., Luna A., Souza E., Fidel S. (2013). Similar sealability between bioceramic putty ready-to-use repair cement and white MTA. Braz. Dent. J..

[100] Toia C.C., Teixeira F.B., Cucco C., Valera M.C., Cavalcanti B.N. (2020). Filling ability of three bioceramic root-end filling materials: A micro-computed tomography analysis. Aust. Endod. J..

[101] Chan S., Glickman G.N., Woodmansey K.F., He J. (2020). Retrospective Analysis of Root-end Microsurgery Outcomes in a Postgraduate Program in Endodontics Using Calcium Silicate-based Cements as Root-end Filling Materials. J. Endod..

[102] Shinbori N., Grama A.M., Patel Y., Woodmansey K., He J. (2015). Clinical outcome of endodontic microsurgery that uses EndoSequence BC root repair material as the root-end filling material. J. Endod..

[103] von Arx T., Janner S.F.M., Haenni S., Bornstein M.M. (2020). Bioceramic root repair material (BCRRM) for root-end obturation in apical surgery. An analysis of 174 teeth after 1 year. Swiss. Dent. J..

[104] Zhou W., Zheng Q., Tan X., Song D., Zhang L., Huang D. (2017). Comparison of Mineral Trioxide Aggregate and iRoot BP Plus Root Repair Material as Root-end Filling Materials in Endodontic Microsurgery: A Prospective Randomized Controlled Study. J. Endod..

[105] Ma X., Li C., Jia L., Wang Y., Liu W., Zhou X., Johnson T.M., Huang D. (2016). Materials for retrograde filling in root canal therapy. Cochrane Database Syst. Rev..

[106] Ayup H., Duane B. (2018). Limited evidence on best material for retrograde root fillings. Evid. Based. Dent..

[107] Plotino G., Abella Sans F., Duggal M.S., Grande N.M., Krastl G., Nagendrababu V., Gambarini G. (2021). European Society of Endodontology position statement: Surgical extrusion, intentional replantation and tooth autotransplantation: European Society of Endodontology developed by. Int. Endod. J..

[108] Becker B.D. (2018). Intentional Replantation Techniques: A Critical Review. J. Endod..

[109] Torabinejad M., Dinsbach N.A., Turman M., Handysides R., Bahjri K., White S.N. (2015). Survival of Intentionally Replanted Teeth and Implant-supported Single Crowns: A Systematic Review. J. Endod..

[110] Cunliffe J., Ayub K., Darcey J., Foster-Thomas E. (2020). Intentional replantation—A clinical review of cases undertaken at a major UK dental school. Br. Dent. J..

[111] Zhang J., Luo N., Miao D., Ying X., Chen Y. (2020). Intentional replantation of periodontally involved hopeless teeth: A case series study. Clin. Oral Investig..

[112] Chogle S., Chatha N., Bukhari S. (2019). Intentional Replantation of Teeth is a Viable and Cost-effective Alternative Treatment to Single-Tooth Implants. J. Evid. Based Dent. Pract..

[113] Mainkar A. (2017). A Systematic Review of the Survival of Teeth Intentionally Replanted with a Modern Technique and Cost-effectiveness Compared with Single-tooth Implants. J. Endod..

[114] Devji T. (2018). Uncertainty about survival rates and cost-effectiveness of intentional replantation for persistent apical periodontitis. J. Am. Dent. Assoc..

[115] Plotino G., Abella Sans F., Bastos J.V., Nagendrababu V. (2022). Effectiveness of intentional replantation in managing teeth with apical periodontitis: A systematic review. Int. Endod. J..

[116] Wang L., Jiang H., Bai Y., Luo Q., Wu H., Liu H. (2020). Clinical outcomes after intentional replantation of permanent teeth: A systematic review. Bosn. J. Basic Med. Sci..

[117] Jang Y., Lee S.J., Yoon T.C., Roh B.D., Kim E. (2016). Survival Rate of Teeth with a C-shaped Canal after Intentional Replantation: A Study of 41 Cases for up to 11 Years. J. Endod..

[118] Cho S.Y., Lee Y., Shin S.J., Kim E., Jung I.Y., Friedman S., Lee S.J. (2016). Retention and Healing Outcomes after Intentional Replantation. J. Endod..

[119] Grzanich D., Rizzo G., Silva R.M. (2017). Saving Natural Teeth: Intentional Replantation-Protocol and Case Series. J. Endod..

[120] Asgary S., Talebzadeh B. (2019). Intentional replantation of a molar with several endodontic complications. J. Stomatol. Oral Maxillofac. Surg..

[121] Asgary S., Nosrat A. (2014). Concurrent intentional replantation of maxillary molars using a novel root-end filling. Gen. Dent..

[122] Kheirieh S., Fazlyab M., Torabzadeh H., Eghbal M.J. (2014). Extraoral Retrograde Root Canal Filling of an Orthodontic-induced External Root Resorption Using CEM Cement. Iran. Endod. J..

[123] Asgary S., Alim Marvasti L., Kolahdouzan A. (2014). Indications and case series of intentional replantation of teeth. Iran. Endod. J..

[124] Karamifar K., Tondari A., Saghiri M.A. (2020). Endodontic Periapical Lesion: An Overview on the Etiology, Diagnosis and Current Treatment Modalities. Eur. Endod. J..

[125] Yang X.Q., Yang R.Q., Tian J., Wei X. (2022). Application status and prospect of single-cone obturation technique with bioceramic sealers. Chin. J. Stomatol..

[126] Coaguila-Llerena H., Ordinola-Zapata R., Staley C., Dietz M., Chen R., Faria G. (2022). Multispecies biofilm removal by a multisonic irrigation system in mandibular molars. Int. Endod. J..

[127] Coaguila-Llerena H., Gaeta E., Faria G. (2022). Outcomes of the GentleWave system on root canal treatment: A narrative review. Restor. Dent. Endod..

[128] Zanza A., Seracchiani M., Reda R., Miccoli G., Testarelli L., Di Nardo D. (2022). Metallurgical Tests in Endodontics: A Narrative Review. Bioengineering.

[129] Heran J., Khalid S., Albaaj F., Tomson P.L., Camilleri J. (2019). The single cone obturation technique with a modified warm filler. J. Dent..

[130] Santos-Junior A.O., Tanomaru-Filho M., Pinto J.C., Tavares K., Torres F.F.E., Guerreiro-Tanomaru J.M. (2021). Effect of obturation technique using a new bioceramic sealer on the presence of voids in flattened root canals. Braz. Oral Res..

[131] Girelli C.F., Lacerda M.F., Lemos C.A., Amaral M.R., Lima C.O., Silveira F.F., Nunes E. (2022). The thermoplastic techniques or single-cone technique on the quality of root canal filling with tricalcium silicate-based sealer: An integrative review. J. Clin. Exp. Dent..

[132] Mekhdieva E., Del Fabbro M., Alovisi M., Comba A., Scotti N., Tumedei M., Carossa M., Berutti E., Pasqualini D. (2021). Postoperative Pain following Root Canal Filling with Bioceramic vs. Traditional Filling Techniques: A Systematic Review and Meta-Analysis of Randomized Controlled Trials. J. Clin. Med..

[133] Aslan T., Dönmez Özkan H. (2021). The effect of two calcium silicate-based and one epoxy resin-based root canal sealer on postoperative pain: A randomized controlled trial. Int. Endod. J..

[134] Chybowski E.A., Glickman G.N., Patel Y., Fleury A., Solomon E., He J. (2018). Clinical Outcome of Non-Surgical Root Canal Treatment Using a Single-cone Technique with Endosequence Bioceramic Sealer: A Retrospective Analysis. J. Endod..

[135] AlBakhakh B., Al-Saedi A., Al-Taee R., Nahidh M. (2022). Rapid Apical Healing with Simple Obturation Technique in Response to a Calcium Silicate-Based Filling Material. Int. J. Dent..

[136] Zavattini A., Knight A., Foschi F., Mannocci F. (2020). Outcome of Root Canal Treatments Using a New Calcium Silicate Root Canal Sealer: A Non-Randomized Clinical Trial. J. Clin. Med..

[137] Bardini G., Casula L., Ambu E., Musu D., Mercadè M., Cotti E. (2021). A 12-month follow-up of primary and secondary root canal treatment in teeth obturated with a hydraulic sealer. Clin. Oral Investig..

[138] Song M., Park M.G., Kwak S.W., Kim R.H., Ha J.H., Kim H.C. (2022). Pilot Evaluation of Sealer-Based Root Canal Obturation Using Epoxy-Resin-Based and Calcium-Silicate-Based Sealers: A Randomized Clinical Trial. Materials.

[139] Cao Y., Bogen G., Lim J., Shon W.J., Kang M.K. (2016). Bioceramic Materials and the Changing Concepts in Vital Pulp Therapy. J. Calif. Dent. Assoc..

[140] Duncan H.F. (2022). Present status and future directions-Vital pulp treatment and pulp preservation strategies. Int. Endod. J..

[141] Ricucci D., Siqueira J.F., Li Y., Tay F.R. (2019). Vital pulp therapy: Histopathology and histobacteriology-based guidelines to treat teeth with deep caries and pulp exposure. J. Dent..

[142] Tong H.J., Seremidi K., Stratigaki E., Kloukos D., Duggal M., Gizani S. (2022). Deep dentine caries management of immature permanent posterior teeth with vital pulp: A systematic review and meta-analysis. J. Dent..

[143] (2021). AAE Position Statement on Vital Pulp Therapy. J. Endod..

[144] Bjørndal L., Simon S., Tomson P.L., Duncan H.F. (2019). Management of deep caries and the exposed pulp. Int. Endod. J..

[145] Ruiz-González P., Cabanillas-Balsera D., Saúco-Márquez J.J., Segura-Egea J.J. (2022). Outcome of Direct Pulp Capping in Teeth Diagnosed as Irreversible Pulpitis: Systematic Review and Meta-Analysis. J. Clin. Exp. Dent..

[146] Matsuura T., Kawata-Matsuura V.K., Yamada S. (2019). Long-term clinical and radiographic evaluation of the effectiveness of direct pulp-capping materials. J. Oral Sci..

[147] Çalışkan M.K., Güneri P. (2017). Prognostic factors in direct pulp capping with mineral trioxide aggregate or calcium hydroxide: 2- to 6-year follow-up. Clin. Oral Investig..

[148] Mente J., Hufnagel S., Leo M., Michel A., Gehrig H., Panagidis D., Saure D., Pfefferle T. (2014). Treatment outcome of mineral trioxide aggregate or calcium hydroxide direct pulp capping: Long-term results. J. Endod..

[149] Kundzina R., Stangvaltaite L., Eriksen H.M., Kerosuo E. (2017). Capping carious exposures in adults: A randomized controlled trial investigating mineral trioxide aggregate versus calcium hydroxide. Int. Endod. J..

[150] Akhlaghi N., Khademi A. (2015). Outcomes of vital pulp therapy in permanent teeth with different medicaments based on review of the literature. Dent. Res. J..

[151] Liu S., Wang S., Dong Y. (2015). Evaluation of a bioceramic as a pulp capping agent in vitro and in vivo. J. Endod..

[152] Mahgoub N., Alqadasi B., Aldhorae K., Assiry A., Altawili Z.M., Tao H. (2019). Comparison between iRoot BP Plus (EndoSequence Root Repair Material) and Mineral Trioxide Aggregate as Pulp-capping Agents: A Systematic Review. J. Int. Soc. Prev. Community Dent..

[153] Motwani N., Ikhar A., Nikhade P., Chandak M., Rathi S., Dugar M., Rajnekar R. (2021). Premixed bioceramics: A novel pulp capping agent. J. Conserv. Dent..

[154] Reis M.S., Scarparo R.K., Signor B., Bolzan J.T., Steier L., Figueiredo J.A.P. (2021). Pulp capping with mineral trioxide aggregate or Biodentine: A comparison of mineralized barrier formation and inflammatory and degenerative events. Braz. Oral Res..

[155] Hoseinifar R., Eskandarizadeh A., Parirokh M., Torabi M., Safarian F., Rahmanian E. (2020). Histological Evaluation of Human Pulp Response to Direct Pulp Capping with MTA, CEM Cement, and Biodentine. J. Dent..

[156] Abdul M.S.M., Murali N., Rai P., Mirza M.B., Salim S., Aparna M., Singh S. (2021). Clinico-Histological Evaluation of Dentino-Pulpal Complex of Direct Pulp Capping Agents: A Clinical Study. J. Pharm. Bioallied. Sci..

[157] Jalan A.L., Warhadpande M.M., Dakshindas D.M. (2017). A comparison of human dental pulp response to calcium hydroxide and Biodentine as direct pulp-capping agents. J. Conserv. Dent..

[158] Cushley S., Duncan H.F., Lappin M.J., Chua P., Elamin A.D., Clarke M., El-Karim I.A. (2021). Efficacy of direct pulp capping for management of cariously exposed pulps in permanent teeth: A systematic review and meta-analysis. Int. Endod. J..

[159] Linu S., Lekshmi M.S., Varunkumar V.S., Sam Joseph V.G. (2017). Treatment Outcome Following Direct Pulp Capping Using Bioceramic Materials in Mature Permanent Teeth with Carious Exposure: A Pilot Retrospective Study. J. Endod..

[160] Parinyaprom N., Nirunsittirat A., Chuveera P., Na Lampang S., Srisuwan T., Sastraruji T., Bua-On P., Simprasert S., Khoipanich I., Sutharaphan T. (2018). Outcomes of Direct Pulp Capping by Using Either ProRoot Mineral Trioxide Aggregate or Biodentine in Permanent Teeth with Carious Pulp Exposure in 6- to 18-Year-Old Patients: A Randomized Controlled Trial. J. Endod..

[161] Brizuela C., Ormeño A., Cabrera C., Cabezas R., Silva C.I., Ramírez V., Mercade M. (2017). Direct Pulp Capping with Calcium Hydroxide, Mineral Trioxide Aggregate, and Biodentine in Permanent Young Teeth with Caries: A Randomized Clinical Trial. J. Endod..

[162] Katge F.A., Patil D.P. (2017). Comparative Analysis of 2 Calcium Silicate-based Cements (Biodentine and Mineral Trioxide Aggregate) as Direct Pulp-capping Agent in Young Permanent Molars: A Split Mouth Study. J. Endod..

[163] Mahmoud S.H., El-Negoly S.A., Zaen El-Din A.M., El-Zekrid M.H., Grawish L.M., Grawish H.M., Grawish M.E. (2018). Biodentine versus mineral trioxide aggregate as a direct pulp capping material for human mature permanent teeth—A systematic review. J. Conserv. Dent..

[164] Zafar K., Nazeer M.R., Ghafoor R., Khan F.R. (2020). Success of pulpotomy in mature permanent teeth with irreversible pulpitis: A systematic review. J. Conserv. Dent..

[165] Sadaf D. (2020). Success of Coronal Pulpotomy in Permanent Teeth with Irreversible Pulpitis: An Evidence-based Review. Cureus.

[166] Coll J.A., Seale N.S., Vargas K., Marghalani A.A., Al Shamali S., Graham L. (2017). Primary Tooth Vital Pulp Therapy: A Systematic Review and Meta-analysis. Pediatr. Dent..

[167] Li Y., Sui B., Dahl C., Bergeron B., Shipman P., Niu L., Chen J., Tay F.R. (2019). Pulpotomy for carious pulp exposures in permanent teeth: A systematic review and meta-analysis. J. Dent..

[168] Linsuwanont P., Wimonsutthikul K., Pothimoke U., Santiwong B. (2017). Treatment Outcomes of Mineral Trioxide Aggregate Pulpotomy in Vital Permanent Teeth with Carious Pulp Exposure: The Retrospective Study. J. Endod..

[169] Alqaderi H.E., Al-Mutawa S.A., Qudeimat M.A. (2014). MTA pulpotomy as an alternative to root canal treatment in children’s permanent teeth in a dental public health setting. J. Dent..

[170] Taha N.A., Ahmad M.B., Ghanim A. (2017). Assessment of Mineral Trioxide Aggregate pulpotomy in mature permanent teeth with carious exposures. Int. Endod. J..

[171] Kang C.M., Sun Y., Song J.S., Pang N.S., Roh B.D., Lee C.Y., Shin Y. (2017). A randomized controlled trial of various MTA materials for partial pulpotomy in permanent teeth. J. Dent..

[172] Taha N.A., Khazali M.A. (2017). Partial Pulpotomy in Mature Permanent Teeth with Clinical Signs Indicative of Irreversible Pulpitis: A Randomized Clinical Trial. J. Endod..

[173] Brignardello-Petersen R. (2017). Mineral trioxide aggregate likely to have a better success rate than calcium hydroxide in mature permanent teeth undergoing partial pulpotomy. J. Am. Dent. Assoc..

[174] Bakhurji E. (2020). Mineral Trioxide Aggregate Could Have a Better Success Rate Than Calcium Hydroxide for Partial Pulpotomy of Symptomatic Mature Permanent Molars. J. Evid. Based Dent. Pract..

[175] Tran X.V., Ngo L.T.Q., Boukpessi T. (2021). Biodentine(TM) Full Pulpotomy in Mature Permanent Teeth with Irreversible Pulpitis and Apical Periodontitis. Healthcare.

[176] Uesrichai N., Nirunsittirat A., Chuveera P., Srisuwan T., Sastraruji T., Chompu-Inwai P. (2019). Partial pulpotomy with two bioactive cements in permanent teeth of 6- to 18-year-old patients with signs and symptoms indicative of irreversible pulpitis: A noninferiority randomized controlled trial. Int. Endod. J..

[177] Çelik B.N., Mutluay M.S., Arıkan V., Sarı Ş. (2019). The evaluation of MTA and Biodentine as a pulpotomy materials for carious exposures in primary teeth. Clin. Oral Investig..

[178] Taha N.A., Abdelkhader S.Z. (2018). Outcome of full pulpotomy using Biodentine in adult patients with symptoms indicative of irreversible pulpitis. Int. Endod. J..

[179] Taha N.A., Abdulkhader S.Z. (2018). Full Pulpotomy with Biodentine in Symptomatic Young Permanent Teeth with Carious Exposure. J. Endod..

[180] Santos J.M., Pereira J.F., Marques A., Sequeira D.B., Friedman S. (2021). Vital Pulp Therapy in Permanent Mature Posterior Teeth with Symptomatic Irreversible Pulpitis: A Systematic Review of Treatment Outcomes. Medicina.

[181] Yang Y., Xia B., Xu Z., Dou G., Lei Y., Yong W. (2020). The effect of partial pulpotomy with iRoot BP Plus in traumatized immature permanent teeth: A randomized prospective controlled trial. Dent. Traumatol..

[182] Azimi S., Fazlyab M., Sadri D., Saghiri M.A., Khosravanifard B., Asgary S. (2014). Comparison of pulp response to mineral trioxide aggregate and a bioceramic paste in partial pulpotomy of sound human premolars: A randomized controlled trial. Int. Endod. J..

[183] Rao Q., Kuang J., Mao C., Dai J., Hu L., Lei Z., Song G., Yuan G. (2020). Comparison of iRoot BP Plus and Calcium Hydroxide as Pulpotomy Materials in Permanent Incisors with Complicated Crown Fractures: A Retrospective Study. J. Endod..

[184] Guan X., Zhou Y., Yang Q., Zhu T., Chen X., Deng S., Zhang D. (2021). Vital Pulp Therapy in Permanent Teeth with Irreversible Pulpitis Caused by Caries: A Prospective Cohort Study. J. Pers. Med..

[185] Nosrat A., Seifi A., Asgary S. (2013). Pulpotomy in caries-exposed immature permanent molars using calcium-enriched mixture cement or mineral trioxide aggregate: A randomized clinical trial. Int. J. Paediatr. Dent..

[186] Asgary S., Eghbal M.J., Bagheban A.A. (2017). Long-term outcomes of pulpotomy in permanent teeth with irreversible pulpitis: A multi-center randomized controlled trial. Am. J. Dent..

[187] Asgary S., Eghbal M.J., Shahravan A., Saberi E., Baghban A.A., Parhizkar A. (2021). Outcomes of root canal therapy or full pulpotomy using two endodontic biomaterials in mature permanent teeth: A randomized controlled trial. Clin. Oral Investig..

[188] Asgary S., Eghbal M.J. (2010). The effect of pulpotomy using a calcium-enriched mixture cement versus one-visit root canal therapy on postoperative pain relief in irreversible pulpitis: A randomized clinical trial. Odontology.

[189] Eghbal M.J., Haeri A., Shahravan A., Kazemi A., Moazami F., Mozayeni M.A., Saberi E., Samiei M., Vatanpour M., Akbarzade Baghban A. (2020). Postendodontic Pain after Pulpotomy or Root Canal Treatment in Mature Teeth with Carious Pulp Exposure: A Multicenter Randomized Controlled Trial. Pain Res. Manag..

[190] Asgary S., Eghbal M.J. (2013). Treatment outcomes of pulpotomy in permanent molars with irreversible pulpitis using biomaterials: A multi-center randomized controlled trial. Acta Odontol. Scand..

[191] Asgary S., Eghbal M.J., Ghoddusi J., Yazdani S. (2013). One-year results of vital pulp therapy in permanent molars with irreversible pulpitis: An ongoing multicenter, randomized, non-inferiority clinical trial. Clin. Oral Investig..

[192] Asgary S., Eghbal M.J., Ghoddusi J. (2014). Two-year results of vital pulp therapy in permanent molars with irreversible pulpitis: An ongoing multicenter randomized clinical trial. Clin. Oral Investig..

[193] Asgary S., Eghbal M.J., Fazlyab M., Baghban A.A., Ghoddusi J. (2015). Five-year results of vital pulp therapy in permanent molars with irreversible pulpitis: A non-inferiority multicenter randomized clinical trial. Clin. Oral Investig..

[194] Bossù M., Iaculli F., Di Giorgio G., Salucci A., Polimeni A., Di Carlo S. (2020). Different Pulp Dressing Materials for the Pulpotomy of Primary Teeth: A Systematic Review of the Literature. J. Clin. Med..

[195] Chen Y., Chen X., Zhang Y., Zhou F., Deng J., Zou J., Wang Y. (2019). Materials for pulpotomy in immature permanent teeth: A systematic review and meta-analysis. BMC Oral Health.

[196] Li W., Mao M., Hu N., Wang J., Huang J., Gu S. (2021). In vitro evaluation of periapical lesion-derived stem cells for dental pulp tissue engineering. FEBS Open Bio..

[197] Liu Y., Gan L., Cui D.X., Yu S.H., Pan Y., Zheng L.W., Wan M. (2021). Epigenetic regulation of dental pulp stem cells and its potential in regenerative endodontics. World. J. Stem. Cells.

[198] Lee H.N., Liang C., Liao L., Tian W.D. (2021). Advances in Research on Stem Cell-Based Pulp Regeneration. Tissue Eng. Regen. Med..

[199] Xie Y., Lu F., Hong Y., He J., Lin Y. (2021). Revascularisation versus apexification for treatment of immature teeth based on periapical healing and root development: A systematic review and meta-analysis. Eur. J. Paediatr. Dent..

[200] Anthrayose P., Nawal R.R., Yadav S., Talwar S., Yadav S. (2021). Effect of revascularisation and apexification procedures on biomechanical behaviour of immature maxillary central incisor teeth: A three-dimensional finite element analysis study. Clin. Oral Investig..

[201] Pereira A.C., Oliveira M.L., Cerqueira-Neto A., Vargas-Neto J., Nagata J.Y., Gomes B., Ferraz C.C.R., de Almeida J.F.A., de-Jesus-Soares A. (2021). Outcomes of traumatised immature teeth treated with apexification or regenerative endodontic procedure: A retrospective study. Aust. Endod. J..

[202] Nicoloso G.F., Goldenfum G.M., Pizzol T., Scarparo R.K., Montagner F., de Almeida Rodrigues J., Casagrande L. (2019). Pulp Revascularization or Apexification for the Treatment of Immature Necrotic Permanent Teeth: Systematic Review and Meta-Analysis. J. Clin. Pediatr. Dent..

[203] Xie Z., Shen Z., Zhan P., Yang J., Huang Q., Huang S., Chen L., Lin Z. (2021). Functional Dental Pulp Regeneration: Basic Research and Clinical Translation. Int. J. Mol. Sci..

[204] Guerrero F., Mendoza A., Ribas D., Aspiazu K. (2018). Apexification: A systematic review. J. Conserv. Dent..

[205] Lin J.C., Lu J.X., Zeng Q., Zhao W., Li W.Q., Ling J.Q. (2016). Comparison of mineral trioxide aggregate and calcium hydroxide for apexification of immature permanent teeth: A systematic review and meta-analysis. J. Formos. Med. Assoc..

[206] Bonte E., Beslot A., Boukpessi T., Lasfargues J.J. (2015). MTA versus Ca(OH)2 in apexification of non-vital immature permanent teeth: A randomized clinical trial comparison. Clin. Oral Investig..

[207] Bücher K., Meier F., Diegritz C., Kaaden C., Hickel R., Kühnisch J. (2016). Long-term outcome of MTA apexification in teeth with open apices. Quintessence Int..

[208] Torabinejad M., Parirokh M., Dummer P.M.H. (2018). Mineral trioxide aggregate and other bioactive endodontic cements: An updated overview—Part II: Other clinical applications and complications. Int. Endod. J..

[209] Ürkmez E., Pınar Erdem A. (2020). Bioactivity evaluation of calcium silicate-based endodontic materials used for apexification. Aust. Endod. J..

[210] Vidal K., Martin G., Lozano O., Salas M., Trigueros J., Aguilar G. (2016). Apical Closure in Apexification: A Review and Case Report of Apexification Treatment of an Immature Permanent Tooth with Biodentine. J. Endod..

[211] Darak P., Likhitkar M., Goenka S., Kumar A., Madale P., Kelode A. (2020). Comparative evaluation of fracture resistance of simulated immature teeth and its effect on single visit apexification versus complete obturation using MTA and biodentine. J. Family Med. Prim. Care.

[212] Bajwa N.K., Jingarwar M.M., Pathak A. (2015). Single Visit Apexification Procedure of a Traumatically Injured Tooth with a Novel Bioinductive Material (Biodentine). Int. J. Clin. Pediatr. Dent..

[213] Niranjan B., Shashikiran N.D., Dubey A., Singla S., Gupta N. (2016). Biodentine-A New Novel Bio-Inductive Material For Treatment of Traumatically Injured Tooth (Single Visit Apexification). J. Clin. Diagn Res..

[214] Nayak G., Hasan M.F. (2014). Biodentine-a novel dentinal substitute for single visit apexification. Restor. Dent. Endod..

[215] Sinha N., Singh B., Patil S. (2014). Cone beam-computed topographic evaluation of a central incisor with an open apex and a failed root canal treatment using one-step apexification with Biodentine™: A case report. J. Conserv. Dent..

[216] Tolibah Y.A., Kouchaji C., Lazkani T., Ahmad I.A., Baghdadi Z.D. (2022). Comparison of MTA versus Biodentine in Apexification Procedure for Nonvital Immature First Permanent Molars: A Randomized Clinical Trial. Children.

[217] Juez M., Ballester M.L., Berástegui E. (2019). In vitro comparison of apical microleakage by spectrophotometry in simulated apexification using White Mineral Trioxide Aggregate, TotalFill Bioceramic Root Repair material, and BioDentine. J. Conserv. Dent..

[218] Pereira I.R., Carvalho C., Paulo S., Martinho J.P., Coelho A.S., Paula A.B., Marto C.M., Carrilho E., Botelho M.F., Abrantes A.M. (2021). Apical Sealing Ability of Two Calcium Silicate-Based Sealers Using a Radioactive Isotope Method: An In Vitro Apexification Model. Materials.

[219] Sockalingam S., Awang Talip M., Zakaria A.S.I. (2018). Maturogenesis of an Immature Dens Evaginatus Nonvital Premolar with an Apically Placed Bioceramic Material (EndoSequence Root Repair Material®): An Unexpected Finding. Case Rep. Dent..

[220] Shaik I., Dasari B., Kolichala R., Doos M., Qadri F., Arokiyasamy J.L., Tiwari R.V.C. (2021). Comparison of the Success Rate of Mineral Trioxide Aggregate, Endosequence Bioceramic Root Repair Material, and Calcium Hydroxide for Apexification of Immature Permanent Teeth: Systematic Review and Meta-Analysis. J. Pharm. Bioallied. Sci..

[221] Lin J., Zeng Q., Wei X., Zhao W., Cui M., Gu J., Lu J., Yang M., Ling J. (2017). Regenerative Endodontics Versus Apexification in Immature Permanent Teeth with Apical Periodontitis: A Prospective Randomized Controlled Study. J. Endod..

[222] Wigler R., Kaufman A.Y., Lin S., Steinbock N., Hazan-Molina H., Torneck C.D. (2013). Revascularization: A treatment for permanent teeth with necrotic pulp and incomplete root development. J. Endod..

[223] El Ashiry E.A., Farsi N.M., Abuzeid S.T., El Ashiry M.M., Bahammam H.A. (2016). Dental Pulp Revascularization of Necrotic Permanent Teeth with Immature Apices. J. Clin. Pediatr. Dent..

[224] Staffoli S., Plotino G., Nunez Torrijos B.G., Grande N.M., Bossù M., Gambarini G., Polimeni A. (2019). Regenerative Endodontic Procedures Using Contemporary Endodontic Materials. Materials.

[225] Li L., Pan Y., Mei L., Li J. (2017). Clinical and Radiographic Outcomes in Immature Permanent Necrotic Evaginated Teeth Treated with Regenerative Endodontic Procedures. J. Endod..

[226] Hameed M.H., Gul M., Ghafoor R., Badar S.B. (2019). Management of immature necrotic permanent teeth with regenerative endodontic procedures—A review of literature. J. Pak. Med. Assoc..

[227] Wongwatanasanti N., Jantarat J., Sritanaudomchai H., Hargreaves K.M. (2018). Effect of Bioceramic Materials on Proliferation and Odontoblast Differentiation of Human Stem Cells from the Apical Papilla. J. Endod..

[228] Wattanapakkavong K., Srisuwan T. (2019). Release of Transforming Growth Factor Beta 1 from Human Tooth Dentin after Application of Either ProRoot MTA or Biodentine as a Coronal Barrier. J. Endod..

[229] Chung S.Y., Kim Y.H., Chae Y.K., Jo S.S., Choi S.C., Nam O.H. (2021). Void characteristics and tortuosity of calcium silicate-based cements for regenerative endodontics: A micro-computed tomography analysis. BMC Oral Health.

[230] Topçuoğlu G., Topçuoğlu H.S. (2016). Regenerative Endodontic Therapy in a Single Visit Using Platelet-rich Plasma and Biodentine in Necrotic and Asymptomatic Immature Molar Teeth: A Report of 3 Cases. J. Endod..

[231] Aldakak M.M., Capar I.D., Rekab M.S., Abboud S. (2016). Single-Visit Pulp Revascularization of a Nonvital Immature Permanent Tooth Using Biodentine. Iran. Endod. J..

[232] Ambu E., Caruso S., Gatto R., Tecco S., Severino M. (2020). Regenerative endodontics procedure of an immature permanent mandibular molar with a necrotic pulp using biodentine: A 16 months radiographic follow-up. J. Biol. Regul. Homeost. Agents.

[233] Cymerman J.J., Nosrat A. (2020). Regenerative Endodontic Treatment as a Biologically Based Approach for Non-Surgical Retreatment of Immature Teeth. J. Endod..

[234] Bukhari S., Kohli M.R., Setzer F., Karabucak B. (2016). Outcome of Revascularization Procedure: A Retrospective Case Series. J. Endod..

[235] Adnan S., Ullah R. (2018). Top-cited Articles in Regenerative Endodontics: A Bibliometric Analysis. J. Endod..

[236] Saed S.M., Ashley M.P., Darcey J. (2016). Root perforations: Aetiology, management strategies and outcomes. The hole truth. Br. Dent. J..

[237] Estrela C., Decurcio D.A., Rossi-Fedele G., Silva J.A., Guedes O.A., Borges Á.H. (2018). Root perforations: A review of diagnosis, prognosis and materials. Braz. Oral Res..

[238] Pinheiro L.S., Kopper P.M.P., Quintana R.M., Scarparo R.K., Grecca F.S. (2021). Does MTA provide a more favourable histological response than other materials in the repair of furcal perforations? A systematic review. Int. Endod. J..

[239] Abboud K.M., Abu-Seida A.M., Hassanien E.E., Tawfik H.M. (2021). Biocompatibility of NeoMTA Plus® versus MTA Angelus as delayed furcation perforation repair materials in a dog model. BMC Oral Health.

[240] Dastorani M., Shourvarzi B., Nojoumi F., Ajami M. (2021). Comparison of Bacterial Microleakage of Endoseal MTA Sealer and Pro-Root MTA in Root Perforation. J. Dent..

[241] de Sousa Reis M., Scarparo R.K., Steier L., de Figueiredo J.A.P. (2019). Periradicular inflammatory response, bone resorption, and cementum repair after sealing of furcation perforation with mineral trioxide aggregate (MTA Angelus™) or Biodentine™. Clin. Oral Investig..

[242] Silva L.A.B., Pieroni K., Nelson-Filho P., Silva R.A.B., Hernandéz-Gatón P., Lucisano M.P., Paula-Silva F.W.G., de Queiroz A.M. (2017). Furcation Perforation: Periradicular Tissue Response to Biodentine as a Repair Material by Histopathologic and Indirect Immunofluorescence Analyses. J. Endod..

[243] Makhlouf M., Zogheib C., Makhlouf A.C., Kaloustian M.K., El Hachem C., Habib M. (2020). Sealing Ability of Calcium Silicate-based Materials in the Repair of Furcal Perforations: A Laboratory Comparative Study. J. Contemp. Dent. Pract..

[244] Mohan D., Singh A.K., Kuriakose F., Malik R., Joy J., John D. (2021). Evaluation of Sealing Potential of Different Repair Materials in Furcation Perforations Using Dye Penetration: An In Vitro Study. J. Contemp. Dent. Pract..

[245] Katge F.A., Shivasharan P.R., Patil D. (2016). Sealing ability of mineral trioxide aggregate Plus™ and Biodentine™ for repair of furcal perforation in primary molars: An in vitro study. Contemp. Clin. Dent..

[246] Sinkar R.C., Patil S.S., Jogad N.P., Gade V.J. (2015). Comparison of sealing ability of ProRoot MTA, RetroMTA, and Biodentine as furcation repair materials: An ultraviolet spectrophotometric analysis. J. Conserv. Dent..

[247] Aslan T., Esim E., Üstün Y., Dönmez Özkan H. (2021). Evaluation of Stress Distributions in Mandibular Molar Teeth with Different Iatrogenic Root Perforations Repaired with Biodentine or Mineral Trioxide Aggregate: A Finite Element Analysis Study. J. Endod..

[248] Kakani A.K., Veeramachaneni C. (2020). Sealing ability of three different root repair materials for furcation perforation repair: An in vitro study. J. Conserv. Dent..

[249] Koç C., Aslan B., Ulusoy Z., Oruçoğlu H. (2021). Sealing ability of three different materials to repair furcation perforations using computerized fluid filtration method. J. Dent. Res. Dent. Clin. Dent. Prospects..

[250] Nazari Moghadam K., Aghili H., Rashed Mohassel A., Zahedpasha S., Moghadamnia A.A. (2014). A comparative study on sealing ability of mineral trioxide aggregate, calcium enriched cement and bone cement in furcal perforations. Minerva Stomatol..

[251] Haghgoo R., Arfa S., Asgary S. (2013). Microleakage of CEM Cement and ProRoot MTA as Furcal Perforation Repair Materials in Primary Teeth. Iran. Endod. J..

[252] Ramazani N., Sadeghi P. (2016). Bacterial Leakage of Mineral Trioxide Aggregate, Calcium-Enriched Mixture and Biodentine as Furcation Perforation Repair Materials in Primary Molars. Iran. Endod. J..

[253] Abdelmotelb M.A., Gomaa Y.F., Khattab N.M.A., Elheeny A.A.H. (2021). Premixed bioceramics versus mineral trioxide aggregate in furcal perforation repair of primary molars: In vitro and in vivo study. Clin. Oral Investig..

[254] Gorni F.G., Andreano A., Ambrogi F., Brambilla E., Gagliani M. (2016). Patient and Clinical Characteristics Associated with Primary Healing of Iatrogenic Perforations after Root Canal Treatment: Results of a Long-term Italian Study. J. Endod..

[255] Mente J., Leo M., Panagidis D., Saure D., Pfefferle T. (2014). Treatment outcome of mineral trioxide aggregate: Repair of root perforations-long-term results. J. Endod..

[256] Rabinovich I.M., Snegirev M.V., Markheev C.I. (2019). Dental root resorption etiology, diagnosis and treatment. Stomatologiia.

[257] Michel A., Erber R., Frese C., Gehrig H., Saure D., Mente J. (2017). In vitro evaluation of different dental materials used for the treatment of extensive cervical root defects using human periodontal cells. Clin. Oral Investig..

[258] Yadav N., Kumar A. (2020). Palatoradicular groove: The hidden predator and etiological factor—Advanced proposed classification and literature review. Indian J. Dent. Res..

[259] Lee K.W., Lee E.C., Poon K.Y. (1968). Palato-gingival grooves in maxillary incisors. A possible predisposing factor to localised periodontal disease. Br. Dent. J..

[260] Kim H.J., Choi Y., Yu M.K., Lee K.W., Min K.S. (2017). Recognition and management of palatogingival groove for tooth survival: A literature review. Restor. Dent. Endod..

[261] Narmatha V.J., Thakur S., Shetty S., Bali P.K. (2014). The complex radicular groove: Interdisciplinary management with mineral trioxide aggregate and bone substitute. J. Contemp. Dent. Pract..

[262] Miao H., Chen M., Otgonbayar T., Zhang S.S., Hou M.H., Wu Z., Wang Y.L., Wu L.G. (2015). Papillary reconstruction and guided tissue regeneration for combined periodontal-endodontic lesions caused by palatogingival groove and additional root: A case report. Clin. Case Rep..

[263] Mittal M., Vashisth P., Arora R., Dwivedi S. (2013). Combined endodontic therapy and periapical surgery with MTA and bone graft in treating palatogingival groove. BMJ Case Rep..

[264] Naik M., de Ataide Ide N., Fernandes M., Lambor R. (2014). Treatment of combined endodontic: Periodontic lesion by sealing of palato-radicular groove using biodentine. J. Conserv. Dent..

[265] Nadig P.P., Agrawal I.S., Agrawal V.S., Srinivasan S.C. (2016). Palato-Radicular Groove: A Rare Entity in Maxillary Central Incisor Leading To Endo-Perio Lesion. J. Clin. Diagn. Res..

[266] Sharma S., Deepak P., Vivek S., Ranjan Dutta S. (2015). Palatogingival Groove: Recognizing and Managing the Hidden Tract in a Maxillary Incisor: A Case Report. J. Int. Oral Health.

[267] Johns D.A., Shivashankar V.Y., Shobha K., Johns M. (2014). An innovative approach in the management of palatogingival groove using Biodentine™ and platelet-rich fibrin membrane. J. Conserv. Dent..

[268] Yan H., Xu N., Wang H., Yu Q. (2019). Intentional Replantation with a 2-segment Restoration Method to Treat Severe Palatogingival Grooves in the Maxillary Lateral Incisor: A Report of 3 Cases. J. Endod..

[269] Xuelian T., Lan Z., Dingming H. (2017). Intentional replantation for the treatment of palatal radicular groove with endo-periodontal lesion in the maxillary lateral incisor: A case report. West China J. Stomatol..

[270] Aidos H., Diogo P., Santos J.M. (2018). Root Resorption Classifications: A Narrative Review and a Clinical Aid Proposal for Routine Assessment. Eur. Endod. J..

[271] Abbott P.V., Lin S. (2022). Tooth resorption-Part 2: A clinical classification. Dent. Traumatol..

[272] Patel S., Foschi F., Condon R., Pimentel T., Bhuva B. (2018). External cervical resorption: Part 2—Management. Int. Endod. J..

[273] Arnold M. (2021). Reparative Endodontic Treatment of a Perforating Internal Inflammatory Root Resorption: A Case Report. J. Endod..

[274] Abuabara A., Costa R.G., Morais E.C., Furuse A.Y., Gonzaga C.C., Filho F.B. (2013). Prosthetic rehabilitation and management of an MTA-treated maxillary central incisor with root perforation and severe internal resorption. J. Prosthodont..

[275] Machado R., Agnoletto M., Engelke Back E.D., Tomazinho L.F., Paganini F.A., Vansan L.P. (2017). Surgical resolution of an aggressive iatrogenic root perforation in a maxillary central incisor: A case report with a 4-year follow-up. Gen. Dent..

[276] Büttel L., Weiger R., Krastl G. (2013). Repair of a root perforation with MTA: A case report. Schweiz. Monatsschr. Zahnmed..

[277] Froughreyhani M., Salem Milani A., Barakatein B., Shiezadeh V. (2013). Treatment of Strip Perforation Using Root MTA: A Case Report. Iran. Endod. J..

[278] Subay R.K., Subay M.O., Tuzcu S.B. (2018). Endodontic management of root perforating internal replacement resorption. Eur. J. Dent..

[279] Bendyk-Szeffer M., Łagocka R., Trusewicz M., Lipski M., Buczkowska-Radlińska J. (2015). Perforating internal root resorption repaired with mineral trioxide aggregate caused complete resolution of odontogenic sinus mucositis: A case report. J. Endod..

[280] Abdullah D., Eziana Hussein F., Abd Ghani H. (2017). Management of Perforating Idiopathic Internal Root Resorption. Iran. Endod. J..

[281] Khalil W.A., Alghamdi F., Aljahdali E. (2020). Strengthening effect of bioceramic cement when used to repair simulated internal resorption cavities in endodontically treated teeth. Dent. Med. Probl..

[282] Aktemur Türker S., Uzunoğlu E., Deniz Sungur D., Tek V. (2018). Fracture Resistance of Teeth with Simulated Perforating Internal Resorption Cavities Repaired with Different Calcium Silicate-based Cements and Backfilling Materials. J. Endod..

[283] Karypidou A., Chatzinikolaou I.D., Kouros P., Koulaouzidou E., Economides N. (2016). Management of bilateral invasive cervical resorption lesions in maxillary incisors using a novel calcium silicate-based cement: A case report. Quintessence Int..

[284] Pruthi P.J., Dharmani U., Roongta R., Talwar S. (2015). Management of external perforating root resorption by intentional replantation followed by Biodentine restoration. Dent. Res. J..

[285] Esnaashari E., Pezeshkfar A., Fazlyab M. (2015). Nonsurgical management of an extensive perforative internal root resorption with calcium-enriched mixture cement. Iran. Endod. J..

[286] Asgary S., Eghbal M.J., Mehrdad L., Kheirieh S., Nosrat A. (2014). Surgical management of a failed internal root resorption treatment: A histological and clinical report. Restor. Dent. Endod..

[287] Asgary S., Nosrat A. (2016). Conservative Management of Class 4 Invasive Cervical Root Resorption Using Calcium-enriched Mixture Cement. J. Endod..

[288] Asgary S., Nosrat A., Seifi A. (2011). Management of inflammatory external root resorption by using calcium-enriched mixture cement: A case report. J. Endod..

[289] Asgary S., Fazlyab M. (2015). Surgical repair of invasive cervical root resorption with calcium-enriched mixture cement: A case report. Gen. Dent..

[290] Howait M., Shaker M., Aljuhani H., AlMohnna M. (2021). External Cervical Resorption: A Case Report and Brief Review of the Literature, and Treatment Algorithms. J. Contemp. Dent. Pract..

[291] Dudeja C., Taneja S., Kumari M., Singh N. (2015). An in vitro comparison of effect on fracture strength, pH and calcium ion diffusion from various biomimetic materials when used for repair of simulated root resorption defects. J. Conserv. Dent..

[292] Ruiz-Linares M., de Oliveira Fagundes J., Solana C., Baca P., Ferrer-Luque C.M. (2022). Current status on antimicrobial activity of a tricalcium silicate cement. J. Oral Sci..

[293] Zhao C., Liu W., Zhu M., Wu C., Zhu Y. (2022). Bioceramic-based scaffolds with antibacterial function for bone tissue engineering: A review. Bioact. Mater..

[294] Zhao Y., Wee C.Y., Zhang H., Yang Z., Wang W.E.J., Thian E.S. (2022). Silver-substituted hydroxyapatite inhibits Pseudomonas aeruginosa outer membrane protein F: A potential antibacterial mechanism. Mater. Sci. Eng. C Mater. Biol. Appl..

[295] Nayak V.V., Tovar N., Hacquebord J.H., Duarte S., Panariello B.H.D., Tonon C., Atria P.J., Coelho P.G., Witek L. (2022). Physiochemical and bactericidal activity evaluation: Silver-augmented 3D-printed scaffolds-An in vitro study. J. Biomed. Mater. Res. B Appl. Biomater..

[296] Xu S., Wu Q., Guo Y., Ning C., Dai K. (2021). Copper containing silicocarnotite bioceramic with improved mechanical strength and antibacterial activity. Mater. Sci. Eng. C Mater. Biol. Appl..

[297] Sánchez-Salcedo S., Shruti S., Salinas A.J., Malavasi G., Menabue L., Vallet-Regí M. (2014). In vitro antibacterial capacity and cytocompatibility of SiO_2_-CaO-P_2_O_5_ meso-macroporous glass scaffolds enriched with ZnO. J. Mater. Chem. B.

[298] Jain P., Garg A., Farooq U., Panda A.K., Mirza M.A., Noureldeen A., Darwish H., Iqbal Z. (2021). Preparation and quality by design assisted (Qb-d) optimization of bioceramic loaded microspheres for periodontal delivery of doxycycline hyclate. Saudi J. Biol. Sci..

[299] Marashdeh M., Stewart C., Kishen A., Levesque C., Finer Y. (2021). Drug-Silica Coassembled Particles Improve Antimicrobial Properties of Endodontic Sealers. J. Endod..

[300] Ndayishimiye J., Kumeria T., Popat A., Falconer J.R., Blaskovich M.A.T. (2022). Nanomaterials: The New Antimicrobial Magic Bullet. ACS Infect. Dis..

[301] Liu Q., Lu W.F., Zhai W. (2021). Toward stronger robocast calcium phosphate scaffolds for bone tissue engineering: A mini-review and meta-analysis. Mater. Sci. Eng. C Mater. Biol. Appl..

[302] Zhang J., Deng F., Liu X., Ge Y., Zeng Y., Zhai Z., Ning C., Li H. (2022). Favorable osteogenic activity of iron doped in silicocarnotite bioceramic: In vitro and in vivo Studies. J. Orthop. Translat..

[303] Myat-Htun M., Mohd Noor A.F., Kawashita M., Baba Ismail Y.M. (2022). Tailoring mechanical and in vitro biological properties of calcium–silicate based bioceramic through iron doping in developing future material. J. Mech. Behav. Biomed. Mater..

[304] Qin H., Wei Y., Han J., Jiang X., Yang X., Wu Y., Gou Z., Chen L. (2022). 3D printed bioceramic scaffolds: Adjusting pore dimension is beneficial for mandibular bone defects repair. J. Tissue Eng. Regen. Med..

[305] Yuan X., Xu Y., Lu T., He F., Zhang L., He Q., Ye J. (2022). Enhancing the bioactivity of hydroxyapatite bioceramic via encapsulating with silica-based bioactive glass sol. J. Mech. Behav. Biomed. Mater..

[306] Taymour N., Fahmy A.E., Gepreel M.A.H., Kandil S., El-Fattah A.A. (2022). Improved Mechanical Properties and Bioactivity of Silicate Based Bioceramics Reinforced Poly(ether-ether-ketone) Nanocomposites for Prosthetic Dental Implantology. Polymers.

[307] Ha W.N., Nicholson T., Kahler B., Walsh L.J. (2017). Mineral Trioxide Aggregate-A Review of Properties and Testing Methodologies. Materials.

[308] Guo Y.J., Du T.F., Li H.B., Shen Y., Mobuchon C., Hieawy A., Wang Z.J., Yang Y., Ma J., Haapasalo M. (2016). Physical properties and hydration behavior of a fast-setting bioceramic endodontic material. BMC Oral Health.

[309] Baghdadi I., Zaazou A., Tarboush B.A., Zakhour M., Özcan M., Salameh Z. (2020). Physiochemical properties of a bioceramic-based root canal sealer reinforced with multi-walled carbon nanotubes, titanium carbide and boron nitride biomaterials. J. Mech. Behav. Biomed. Mater..

[310] Jiménez-Sánchez M.C., Segura-Egea J.J., Díaz-Cuenca A. (2020). A Microstructure Insight of MTA Repair HP of Rapid Setting Capacity and Bioactive Response. Materials.

[311] Abdalla M.M., Lung C.Y.K., Neelakantan P., Matinlinna J.P. (2020). A novel, doped calcium silicate bioceramic synthesized by sol-gel method: Investigation of setting time and biological properties. J. Biomed. Mater. Res. B Appl. Biomater..

[312] Sony S., Suresh Babu S., Nishad K.V., Varma H., Komath M. (2015). Development of an injectable bioactive bone filler cement with hydrogen orthophosphate incorporated calcium sulfate. J. Mater. Sci. Mater. Med..

[313] Li Q., Coleman N.J. (2019). Impact of Bi_2_O_3_ and ZrO_2_ Radiopacifiers on the Early Hydration and C-S-H Gel Structure of White Portland Cement. J. Funct. Biomater..

[314] Koju N., Sikder P., Gaihre B., Bhaduri B.S. (2018). Smart Injectable Self-Setting Monetite Based Bioceramics for Orthopedic Applications. Materials.

[315] Donnermeyer D., Schemkämper P., Bürklein S., Schäfer E. (2022). Short and Long-Term Solubility, Alkalizing Effect, and Thermal Persistence of Premixed Calcium Silicate-Based Sealers: AH Plus Bioceramic Sealer vs. Total Fill BC Sealer. Materials.

[316] Chaves de Souza L., Teixeira Neves G.S., Kirkpatrick T., Letra A., Silva R. (2022). Physico-chemical and biological properties of AH Plus Bioceramic. J. Endod..

[317] Silva E., Cardoso M.L., Rodrigues J.P., De-Deus G., Fidalgo T. (2021). Solubility of bioceramic- and epoxy resin-based root canal sealers: A systematic review and meta-analysis. Aust. Endod. J..

[318] Mendes A.T., Silva P.B.D., Só B.B., Hashizume L.N., Vivan R.R., Rosa R.A.D., Duarte M.A.H., Só M.V.R. (2018). Evaluation of Physicochemical Properties of New Calcium Silicate-Based Sealer. Braz. Dent. J..

[319] Placek L.M., Keenan T.J., Coughlan A., Wren A.W. (2022). Synthesis, Processing and the Effect of Thermal Treatment on the Solubility, Antioxidant Potential and Cytocompatibility of Y_2_O_3_ and CeO_2_ doped SiO_2_-SrO-Na_2_O Glass-Ceramics. J. Biomater. Appl..

[320] Baghdadi I., AbuTarboush B., Zaazou A., Skienhe H., Özcan M., Zakhour M., Salameh Z. (2021). Effect of sintering temperature on the physiochemical properties, microstructure, and compressive strength of a bioceramic root canal sealer reinforced with multi-walled carbon nanotubes and titanium carbide. J. Mech. Behav. Biomed. Mater..

